# Kinetic model of GPCR-G protein interactions reveals allokairic modulation of signaling

**DOI:** 10.1038/s41467-022-28789-5

**Published:** 2022-03-08

**Authors:** Kelly J. Culhane, Tejas M. Gupte, Indrani Madhugiri, Chetan J. Gadgil, Sivaraj Sivaramakrishnan

**Affiliations:** 1 Department of Genetics, Cell Biology and Development, University of Minnesota, Twin Cities, Minneapolis, MN 55455 USA; 2 Chemical Engineering Division, CSIR-National Chemical Laboratory, Pune, 411008 India; 3 CSIR-Institute of Genomics and Integrative Biology, New Delhi, 110025 India

**Keywords:** Hormone receptors, Computational models

## Abstract

Established models of ternary complex formation between hormone, G protein coupled receptor (GPCR), and G protein assume that all interactions occur under equilibrium conditions. However, recent studies have established that the lifetimes of these interactions are comparable to the duration of hormone activated GPCR signaling. To simulate interactions during such non-equilibrium conditions, we propose a kinetic model wherein the receptor undergoes rate-limiting transitions between two hormone-bound active states. Simulations, using experimentally measured parameters, demonstrate transient states in ternary complex formation, and delineate the phenomenon of GPCR priming, wherein non-cognate G proteins substantially enhance cognate G protein signaling. Our model reveals that kinetic barriers of slow receptor interconversion can be overcome through allokairic modulation, a regulatory mechanism of ternary complex formation and downstream signaling.

## Abstract (web summary)

Experimentally validated kinetic simulations uncover transient enhancement of GPCR ternary complex formation by allokairic effectors.

## Introduction

In the classic ternary complex model of G protein coupled receptor (GPCR) signaling, the receptor populates binary inactive (R_i_) and active (R_a_) states^1–5^ (Supplementary Fig. 1a). Hormone (H) and G protein synergistically promote the active state to activate downstream signaling. In contrast, structural and spectroscopic studies over the last decade demonstrate that the receptor populates a continuum of conformational states that progressively promote G protein activation^6,7^. The receptor is held in an inactive conformation by interhelical ionic locks that act as molecular switches. Ligand binding enhances receptor conformational heterogeneity, toggling these molecular switches to facilitate receptor activation^8^. The presence of multiple receptor conformational states, each of which can differentially engage ligand and G protein, suggests the need for a continuum model of ternary complex formation (Supplementary Fig. 1b). Further, several studies have highlighted the role of G protein nucleotide state on the kinetics of ligand binding and receptor conformation^9–11^ necessitating the inclusion of G protein activation states in models of GPCR signaling (Supplementary Figs. 2,3).

Classic and continuum models assume that ligand, G protein and receptor interactions are at equilibrium. However, several independent studies have established that timescales of ternary complex association and dissociation are longer than the duration of standard experimental timelines^10,12–14^. Specifically, recent studies of the β2 adrenergic receptor (β2AR) suggests that the formation of the fully-coupled state of the β2AR-Gs complex requires ~100 min^15–17^. Likewise, the fully active state of β2AR can trap the agonist, resulting in ligand-receptor dissociation times > 60 min^10^. Thus, during the short durations (~5 min) probed by live cell downstream second messenger assays^18^, experimental data are likely to correspond to intermediate states and therefore the transient response, rather than a steady-state. Further, equilibrium models are unable to describe the recent phenomenon of priming in β2AR, where the presence of a non-cognate G protein (Gq) increases signaling through cognate (Gs) pathways^13,19^. Priming is a temporal process, where exposure to the C-terminus of the Gαq subunit catalyzes the formation of long-lived conformational states of β2AR (*t*_1/2_ = 90 s)^13^. Such long-lived states are hypothesized to increase Gs interactions, thus increasing cognate signaling^13,19^. Taken together, these observations require consideration of non-equilibrium conditions stemming from rate-limiting transitions in the formation of the ternary complex. Hence, in this study, we developed and experimentally validated a conceptual framework to model transient, non-equilibrium conditions during GPCR signaling.

Our model parallels efforts to delineate receptor-G protein interaction kinetics using extensive single molecule FRET measurements of a β2AR conformational biosensor^11^. This study proposed a multi-step model of receptor and G protein activation, wherein G protein binding is essential for complete receptor activation (Supplementary Fig. 4a). While the agonist bound receptor alone rarely transitioned to its fully active state (~1 min^−1^), the presence of the G protein enhanced this transition rate to 0.1–0.7 s^−1^ in an agonist efficacy dependent manner^11^. These data suggest kinetic barriers between receptor conformational states (HR’ → HR*; Supplementary Fig. 4) that mirror the slow movements in the β2AR transmembrane domains in response to some agonists (~1–3 min^20^) and the metastable active conformational states (lifetime of ~1 min) observed in rhodopsin following ligand dissociation^12^. Despite these advances, a detailed kinetic model that allows incorporation of the effects of non-cognate G protein or other effectors that interface with the receptor is still lacking and forms the emphasis of our study.

Here, we use a combination of experimental and kinetic simulation data to understand the effect of non-equilibrium conditions on G protein-GPCR interactions and signaling downstream of β2AR. We derive a simplified, two-state model, in which the hormone-bound receptor undergoes a rate-limiting transition between two active states (HR’ and HR*). Receptor active states were probed using a FRET-based SPASM sensor that measured interactions between β2AR and the C-terminus of the Gα subunit (G peptide), a major element of the G protein interacting interface. Stopped flow decays of quenching SPASM sensor FRET using a high affinity G protein binding site nanobody (Nb6B9) show two distinct lifetimes for the β2AR interaction with its cognate Gs peptide (Spep), supporting the existence of two receptor-G protein interaction states (HR’G and HR*G). Our model addresses the non-intuitive observation of G protein priming. Under the non-equilibrium timeframe of experimental measurements (~5 min), simulations show that the presence of a weakly binding non-cognate G protein increased ternary complex formation with the cognate G protein. Experimentally, the presence of the non-cognate, Gq peptide (Qpep) increases the cognate β2AR-Spep interaction in pulldown assays, validating the simulation results. Our model reveals that Qpep acts a positive allokairic modulator, which increases cognate ternary complex formation by overcoming the rate limiting conformational transition between receptor interaction states (HR’ → HR*). Allokairic modulation with Qpep yields a disproportionate increase in both β2AR-Gs ternary complex formation and downstream cAMP signaling for a partial compared to a full agonist. Together, our integrated kinetic modeling and experimental data highlight the importance of understanding the timescales of interactions in GPCR signaling pathways, while establishing allokairic regulation in ternary complex formation and downstream signaling.

## Results

**Fig. 1:**
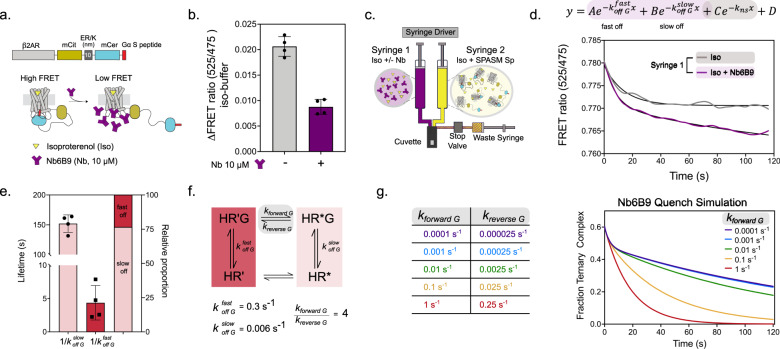
G peptide-receptor interactions show two distinct kinetic profiles. **a** Schematic showing the FRET construct and ΔFRET assay. Nb6B9 binds free receptor (HR’ and HR*) to quench the agonist activated β2AR interaction with Spep in β2AR-Spep SPASM sensors in native membranes prepared from Hek293T cells. **b** Change in FRET ratio (mCit/mCer) with isoproterenol treatment (iso, 100 μM) with and without of 10 μM Nb6B9. Nb6B9 decreases the Spep activation. Data are presented as mean +/- the standard deviation from 4 membrane preparations (*n* = 4). **c** Schematic of the stopped-flow injection set-up used to measure decay in the FRET ratio of agonist activated β2AR-Spep SPASM sensors after mixing with 10 μM Nb6B9. Both syringes contain 100 μM isoproterenol. **d** Representative decay curve of the decrease in FRET ratio over time when syringe 1 contains 100 μM iso (grey) or 100 μM iso + 10 μM Nb6B9 (purple). The iso curve (grey) was fit a single exponential decay with the rate *k*_*ns*_. The Nb6B9 decay (purple) was fit to a triple exponential decay, constraining one decay to the iso fit (see Supplementary Fig 7 for all decay curves and fitting parameters). **e** Lifetime (1/*k*_*off G*_*)* of two states of the Nb6B9 decay and relative proportion of the slow off and fast off states. Data are presented as mean +/− standard deviation from 4 membrane preparations (*n* = 4). **f** Kinetic model with constants constrained by experimentally measured rates. **g** Simulated Nb6B9 quench data show the interconversion rates from HR’G → HR*G must have lifetimes lower than the duration of the decay (see Supplementary Figure 8 for details of modeling). Source data are provided in the Source Data File.

As a simplification of the continuum scenario (the ‘tesseract model’, Supplementary Figs. 2–4), we propose a model with a single, rate-limiting conformational transition of the hormone-bound receptor (HR’ → HR*) between two functionally distinct receptor states (Supplementary Fig. 1c). In this model, transitions between receptor states occur either spontaneously or through a catalyzed process, with the efficacy of the facilitated transition dependent on the G protein or modulator (Supplementary Fig. 5). We hypothesize that the distinct receptor states (HR’ and HR*) interact differently with the G protein, possibly through changes in binding kinetics and affinity. To test this hypothesis, we used a stopped-flow quench assay to measure the off-rate of the C-terminus of the Gα subunit (G-peptide), a key component of the agonist-activated GPCR-G protein interface^21^. We focus on the interaction between agonist-stimulated β2AR and its cognate Gαs-peptide (Spep) using a SPASM sensor^18^ (β2AR-Spep; Fig. 1a). Under steady-state conditions, the addition of a G protein mimetic nanobody (Nb6B9) to β2AR-Spep, displaces the Spep, decreasing the FRET readout of the SPASM sensor (Fig. 1b). Using a stopped flow assay, we measured the decrease over time in FRET signal from β2AR-Spep, preincubated with agonist (100 µM isoproterenol), due to quenching with Nb6B9 (10 µM, Fig. 1c). The FRET signal of β2AR-Spep was quenched faster and more substantially in the presence of Nb6B9 compared to β2AR-Spep alone (Fig. 1d). We observe a single exponential decay in FRET ratio in the absence of Nb6B9 (t_1/2_~15 s) that represents the equilibration time for the mixed crude membrane preparation (Supplementary Fig. 6a). The kinetic FRET profile in the presence of Nb6B9 displays a fast, initial decay phase (<10 s) followed by a sustained decrease over an extended time (10–100 s). Hence, the FRET decay profile was modeled as a triple exponential decay, with one component constrained by the crude membrane equilibration time (Fig. 1d). From this model, we measure two distinct lifetimes of Spep interaction with β2AR (Fig. 1e, Supplementary Fig. 7). Our data reveals a combination of weak (*k*_*off*_ = 0.3 s^−1^) and strong interactions (*k*_*off*_ = 0.006 s^−1^) of β2AR with Spep, with the strong interaction dominating the kinetic profile (75%; Fig. 1e). The different interaction affinities observed are consistent with the presence of two distinct states in the β2AR-Spep interaction.

Essentially, for the stopped flow decay to resolve two distinct lifetimes, the interconversion rates would have to be either less than or equal to the inverse of the longer lifetime (Fig. 1e; 1/150 = 0.007 s^−1^). If this were not the case, the exchange between the receptor states when bound to the Spep would result in the interconversion of the long-lived interaction state to the short-lived one, and we would observe primarily a single exponential decay that reflects the short-lived interaction. Instead, the occurrence of a two-exponential decay with a major component of the long-lived interaction (75%; Fig. 1e) leads us to conclude that the interconversion rate is ≤0.007 s^−1^. This inference is formally demonstrated using our transient kinetic model (Fig. 1f, g). Incorporation of the measured off-rates into our kinetic model (Fig. 1f), recapitulates the experimentally derived FRET profiles (Fig. 1g, Supplementary Fig. 8). Importantly, we find that the simulated kinetic FRET profile is dependent on the rate of interconversion between weak and strong interaction states (Fig. 1g). Specifically, our simulations show that detection of two interaction states requires interconversion rates (*k*_*forward G*_, *k*_*reverse G*_) that are significantly lower than the off-rate (*k*_*off*_) of the strong interaction (Fig. 1g). For fast inter-conversion rates (*k*_*forward*_ > 0.1 s^−1^), the strong interaction is not observed. Instead, the receptor interconverts to the weak interaction state, with subsequent dissociation at rates consistent with the weak interaction (Fig. 1g). The kinetic measurements of two distinct β2AR-Spep interaction states (HR’G and HR*G), combined with simulations that demonstrate the requirement for slow interconversion rates between HR’G and HR*G, provide a strong foundation for our 2-state model as a tool to investigate how changes in receptor-G protein interaction states affect GPCR signaling.

**Fig. 2:**
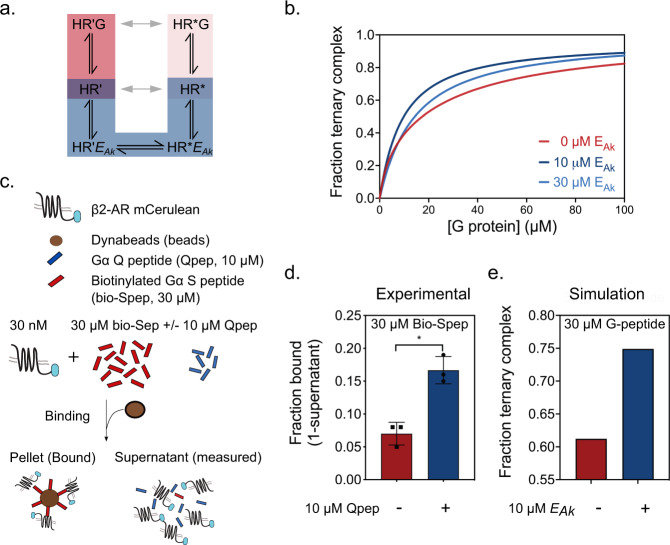
Allokairic Effector (EAk) increases ternary complex formation. **a** Extended model incorporating an allokairic effector (*E*_*Ak*_*)*. **b** Transient simulations show increases in ternary complex as the concentration of G protein increases (red), which is further enhanced by the presence of *E*_*Ak*_ (blue). **c** Schematic of the affinity sequestration assay experimental procedure. **d** 30 μM bio-Spep binds β2AR–mCer in native membranes (red). The addition of 10 μM Qpep acting as *E*_*Ak*_ increases β2AR–mCer binding to bio-Spep 2-fold (blue). Data are presented as the mean +/− standard deviation from 3 independent membrane preparations (*n* = 3) *p = 0.035 in two-sided, paired t-test. **e** Transient simulations show ternary complex with 30 μM G peptide present (red) in the absence and presence of 10 μM *E*_*Ak*_ (blue). Simulation parameters in S Table 1. Source data are provided in the Source Data File.

A key feature in our model is the slow interconversion, relative to experimental duration, between receptor conformational states that have ~50-fold differences in *k*_*off*_ for the G-peptide (Fig. 1f). Hence, factors that accelerate interconversion between these receptor conformational states will impact G-peptide binding strength and consequently efficacy of downstream signaling. We have previously shown that effectors that bind at the G protein binding site, including the non-cognate Gαq-peptide (Qpep), can serve as positive allokairic modulators of the GPCR, by priming the receptor for increased signaling^13,22^. Here, we investigated the kinetic basis of allokairic modulation in the context of an extension of our transient model (Fig. 2a: *extended model*). As expected, tight binding of an allokairic effector (*E*_*Ak*_) to the receptor, relative to the cognate G protein (*K*_*E*_ < *K*_*D*_*),* results in competitive inhibition of ternary complex formation and consequent downstream signaling (Supplementary Fig. 9). This observation is consistent with the theoretical predication that at steady-state, a competing modulator, irrespective of affinity, cannot increase the extent of ternary complex formation (see proof in Supplemental Note). In contrast, our model simulations reveal that weak binding of *E*_*Ak*_, rather than resulting in a weak inhibition, transiently enhances ternary complex formation, at all cognate G protein concentrations that are comparable to or higher than the concentration of *E*_*Ak*_ (Fig. 2b). This result provides a mechanistic explanation for the unintuitive effect of priming, as observed in the non-cognate Gq protein enhancement of cAMP signaling through β2AR-Gs interactions^19^. To experimentally validate the observations from the extended model, we quantified ternary complex formation between β2AR and Gαs-peptide (Spep) in the presence of Gαq peptide (Qpep) serving as *E*_*Ak*_. β2AR-mCerulean (mCer) membranes were incubated with saturating concentrations of agonist (100 μM isoproterenol) and 30 μM N-terminally biotinylated Gαs-peptide (bio-Spep) in the presence or absence of Qpep (Fig. 2c). Streptavidin-coated magnetic beads were used to separate bio-Spep and bound β2AR-mCer membranes. The residual receptor in the supernatant was used to quantify the fraction of receptor bound to bio-Spep (Fig. 2d). The addition of Qpep enhanced the fraction of bound receptor from 5% to 17% (Fig. 2d). This data mirrors the enhanced ternary complex formation, in the presence of *E*_*Ak*_, observed in our extended model using matched concentrations of G protein and *E*_*Ak*_ (Fig. 2e). Together, our experimental and modeling results show that *E*_*Ak*_, despite, and in fact due to, its weak binding, transiently facilitates ternary complex formation with the cognate G protein. Such transient enhancement establishes the utility of the extended model in investigating the non-intuitive temporal regulation of allokairic and other effectors on G protein-GPCR interactions.

**Fig. 3:**
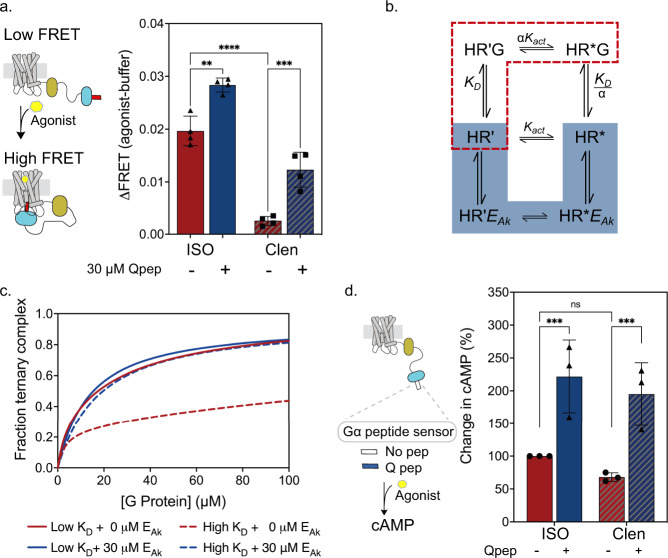
EAk disproportionately increases ternary complex formation for low efficacy agonists. **a** Change in FRET ratio for β2AR-Spep sensors in the presence of high efficacy (100 μM ISO, red) and low efficacy (100 μM clen, red dashed) ligands. Decreased ΔFRET due to clen stimulation is increased with the addition of 30 μM Qpep (blue dashed). Data shows the mean +/- standard deviation from 3 independent membrane preparations of SPASM sensors (*n* = 3) **p = 0.0011, ***p = 0.0003, ****p < 0.0001 in two way ANOVA with Tukey’s multiple comparisons test. **b** Extended model, highlighting G protein binding affinity for HR’G (*K*_*D*_) and HR*G (*K*_*D*_/α), and receptor interconversion constants (*K*_*act*_ and α*K*_*act*_). **c** Lower *K*_*D*_ (*K*_*D*_ = 30 μM, red) increases ternary complex formation compared to high *K*_*D*_ (*K*_*D*_ = 300 μM, red dashed). The addition of 30 μM E_Ak_ increases ternary complex with both low *K*_*D*_ (blue) and high *K*_*D*_ (blue dashed), with a greater effect with high *K*_*D*_ values. *K*_*D/*_α is kept constant through all conditions (*K*_*D*_/α = 0.75 μM). Additional agonist efficacy modeling in Supplementary Fig 10. **d** cAMP accumulation in cells transfected with β2AR-no peptide or β2AR-Qpep SPASM sensors after 5 min of stimulation with either high efficacy (10 μM ISO) or low efficacy (10 μM clen) ligand. Change in cAMP is normalized to ISO with no Qpep as 100%. Data show the mean +/– standard deviation for 3 independent experiments (*n* = 3) ***p = 0.0007 in 2-way ANOVA with Tukey’s multiple comparisons test. Source data are provided in the Source Data File.

Thus far, our experimental observations of GPCR priming have examined the impact of non-cognate G-peptides, acting as *E*_*Ak*_, in the context of a full agonist. A recent study from our lab shows that agonist efficacy linearly correlates with the strength of the interaction between β2AR and Spep^23^. Thus, the partial agonist, clenbuterol, stimulates a 6-fold weaker β2AR-Spep interaction compared to the full agonist, isoproterenol (Fig. 3a). Nonetheless, *E*_*Ak*_ (Qpep) drives a disproportionate increase in the β2AR-Spep interaction for clenbuterol compared to isoproterenol (2-fold for ISO vs 5-fold for Clen; Fig. 3a). To understand the intersection between agonist efficacy and *E*_*Ak*_ in the context of our simplified model (Fig. 3b), we studied the effects of binding parameters (*K*_*D*_, *K*_*D*_ */ α, α*) on ternary complex formation in the presence and absence of *E*_*Ak*_ (Supplementary Fig. 10). Varying the interaction strength of the G protein for HR’ (*K*_*D*_), with or without modulation of its affinity for HR* (*K*_*D*_ */ α*), significantly impacted ternary complex formation in the absence of *E*_*Ak*_ (Supplementary Fig. 10b, d; Fig. 3c). The model prediction of reduced ternary complex formation with higher *K*_*D*_ (lower affinity) is consistent with experimental observations of decreased G protein activation observed with lower agonist efficacy^11,23^. However, increasing *K*_*D*_ alone, without impacting *K*_*D*_ */ α*, results in a disproportionate effect of *E*_*Ak*_ in augmenting ternary complex formation (Supplementary Fig. 10c; Fig. 3c). Regardless, the presence of *E*_*Ak*_ results in a net increase in coupling, by circumventing a kinetic barrier in the formation of a strongly coupled state (HR*G). The presence of *E*_*Ak*_ results in a catalyzed interconversion between receptor conformational states (HR’ → HR*), changing the kinetics of the distribution of receptor-G protein interaction states and transiently enhancing ternary complex formation (Fig. 3b). Hence, our model predicts that *E*_*Ak*_ has the potential to minimize disparities in signaling between partial and full agonists. To test this prediction, we examined cAMP accumulation downstream of β2AR activation in the absence or presence of Qpep (Fig. 3d). The presence of Qpep increases both isoproterenol (full agonist) and clenbuterol (partial agonist) stimulation of cAMP production, with a disproportionate increase for the partial agonist (Fig. 3d). The ability of Qpep, acting as *E*_*Ak*_, to increase downstream signaling in cell-based assays, establishes the cellular relevance of the receptor interaction states in the extended model. Together, our findings provide a conceptual foundation for the ability of *E*_*Ak*_ to facilitate receptor conformational transitions limited by agonist efficacy under non-equilibrium conditions.

## Discussion

**Fig. 4:**
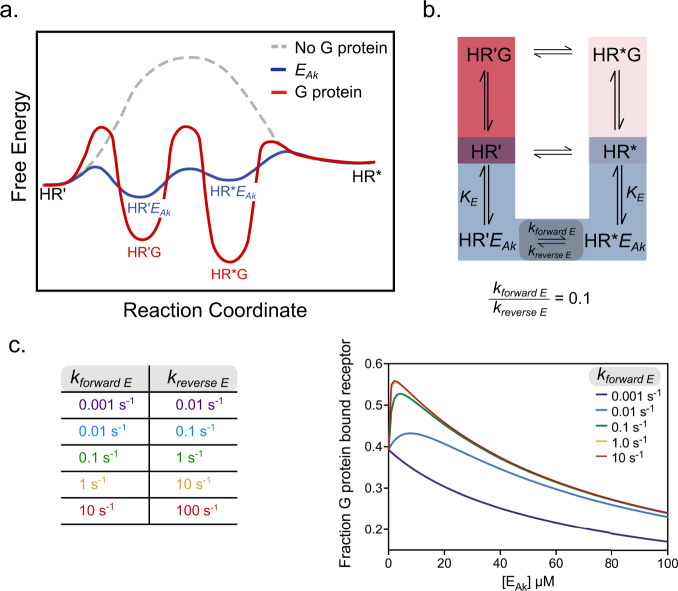
Allokairic modulation decreases the activation barriers for G protein ternary complex formation. **a** Free-energy diagram showing transitions between receptor states. **b** Extended model showing the dissociation (K_E_) of the allokairic effector (*E*_*Ak*_) and the forward and reverse rates for receptor interconversion by *E*_*Ak*_. **c** Simulations varying the speed of receptor interconversion rates with *E*_*Ak*_ present show the interconversion rates determine effect of the *E*_*Ak*_ on ternary complex formation with 10 μM G protein present. Source data are provided in the Source Data File.

In this study, we have developed a model to examine the impact of non-equilibrium conditions on GPCR signaling. Our model incorporates experimental evidence for slow interconversion between receptor conformational states, relative to the duration of faster cellular signaling processes^24,25^ (~mins). This slow interconversion can be viewed from the perspective of an activation barrier between distinct receptor states on a free energy landscape (Fig. 4). Hormone binding facilitates the formation of active receptor conformational states (HR’), with a rate-limiting transition to fully active states (HR*, Fig. 4a). While the G protein binds with significantly higher affinity to HR*, the formation of HR*G is limited by receptor availability (HR*). Further, agonist efficacy tunes G protein affinity for HR’, with consequent impact on ternary complex formation. In the context of this simplified model, positive allokairic modulators (PAkM) serve as allokairic effectors (*E*_*Ak*_) to catalyze the formation of fully active receptor states (HR*). *E*_*Ak*_ binding to the receptor is necessarily weak, to prevent sequestration of the receptor in signaling-incompetent receptor-*E*_*Ak*_ complexes (Supplementary Fig. 9). *E*_*Ak*_ augments ternary complex formation by accelerating the rates of receptor inter-conversion between HR’ and HR*, effectively lowering the activation barrier between the two states (Fig. 4a). The relative effect of *E*_*Ak*_ on ternary complex formation and consequent downstream signaling can be tuned by *E*_*Ak*_ effects on receptor inter-conversion rates (Fig. 4b, c).

Our study outlines a general framework with apo (R) and ligand-bound (HR) states of the receptor, and both inactive (GDP/empty) and active (GTP) states of the G protein (Supplementary Fig. 2). This framework includes both the reversible reactions, (Supplementary Fig. 2) and differential equations for time-varying concentrations (Supplementary Fig. 3), that together describe the time-dependent interconversion of all outlined species (Supplementary Fig. 2). Within this framework, we have focused our computational and experimental efforts to examine the impact of allokairic effectors at saturating ligand concentrations (HR). While equilibrium constants (K_D_/K_A_) for ligand binding to distinct receptor states (low or high affinity) are well documented, association and dissociation constants need to be dissected for incorporation into a transient kinetic model. Such experimental measurements will enable future efforts to examine the effects of allokairic effectors at sub-saturating concentrations that occur in physiological settings. We also simplify our model to consolidate the inactive and active states of the G protein (Supplementary Fig. 4b, c), to focus on rate-limiting events prior to ternary complex formation. Such simplification integrates the events following G protein binding (Supplementary Fig. 4b; *steps 4-5*) into a single rate-limiting event (Supplementary Fig. 4c; *step 4*). The rate of receptor interconversion following G protein activation has been precisely documented (~1 s^−1^; Supplementary Fig. 4b; *steps 5* + *6* ;^11^). However, the nucleotide-dependent G protein association/dissociation rates from the receptor are unclear, in part due to the potential for sustained receptor-G protein engagement. Receptors display a wide range of pre-association or pre-coupling to G proteins prior to ligand activation (GDP bound^26^) and can remain bound to them following G protein activation (GTP bound^27^). Future efforts can expand the model to incorporate receptor-specific effects on G protein engagement in the context of our generalized model (Supplementary Fig. 2).

Our model extends the kinetic framework proposed by Gregorio et al.^11^ to incorporate the influence of effectors that bind at the G protein binding site. Gregorio et al proposed a two-state model of receptor activation, wherein G protein binding was essential for receptor transition to its fully active state (equivalent to R*; Supplementary Fig. 4a), characterized by a low FRET signal in their conformational biosensor. In the absence of G protein, such conformational transitions were infrequent (1 min^−1^; red arrows Supplementary Fig. 4) and comparable to the timescale of downstream second messenger signaling (~5 min). The equilibrium model-equivalent of the Gregorio et al framework includes inactive (GDP or empty) and active (GTP) states of the G protein, with single molecule measurements outlining the dominant kinetic pathway for G protein activation (Supplementary Fig. 4a - *steps 1-7*). In this study, we extend this model to incorporate the influence of an allokairic effector (*E*_*Ak*_) that shares the G protein binding site. *E*_*Ak*_ binds to the hormone-bound receptor (HR’) and rapidly transitions it to a fully active state (HR*; Supplementary Fig. 4b – *steps 1-3*) before dissociating and allowing for G protein coupling (HR*G). Our experimental data (Fig. 1) establish that the fully active receptor (R*) has a significantly higher affinity (lower off-rate) for G protein compared to partially active states (R’; Supplementary Fig. 4b – *red arrow*). Hence, *E*_*Ak*_ enhances ternary complex formation (HR’G + HR*G), subsequent G protein activation (Supplementary Fig. 4b - *steps 5-7*), and hence downstream signaling.

Our model focuses on a rate-limiting conformational transition in the receptor (HR’ → HR*). Several structural and spectroscopic studies have identified fast conformational transitions between intermediate states (~ms^11,28^), that our model groups together in a single state (R’; Supplementary Fig. 1b). These fast conformational transitions are consistent with the general view of GPCRs as highly dynamic structures that rapidly populate a broad conformational landscape following ligand activation^7^. In contrast, the structural basis of the rate-limiting transition that is the focus of this study remains unclear. We speculate on two potential mechanisms that mediate this transition, including cationic stabilization and protein folding. First, both monovalent (Na^+^) and divalent (Ca^2+^) cations are established allosteric modulators of GPCR signaling^29^. Na^+^, in particular, has a well-documented binding site in class A GPCRs that is essential for downstream signaling. The coordinated Na^+^/water cluster embedded within the receptor could selectively stabilize conformational states and influence interconversion kinetics. Second, the interaction of non-cognate G proteins or peptides (Qpep) with the receptor within its cytosolic pocket could facilitate changes in protein folding within the transmembrane helices or the loop regions connecting them. Protein folding/unfolding rates involving single-step folding with α-helices and/or β-sheets have been precisely documented^30^. These rates are found to track linearly with the size of the protein fold^31^ and readily encompass the ~ min scale transitions between conformational states in the absence of G protein^11^ or allokairic effector (Fig. 1).

We focus on allokairic effectors that compete with G protein for receptor binding. Hence, our models delineate mutually exclusive interactions involving G protein (H·R·G) and *E*_*Ak*_ (H·R·*E*_*Ak*_), without their simultaneous engagement to the receptor (H·R·G·*E*_*Ak*_). In contrast, allosteric modulators typically engage the receptor at a site that does not overlap with ligand or G protein binding^32^. Hence, while allosteric modulators demonstrate saturable effects on signaling, the allokairic effectors examined in this study, including the non-cognate G protein/peptides, display enhanced effects at an optimal concentration that is dependent on the kinetics of interconversion between receptor activation states (Fig. 4d). Despite this concentration dependence, we propose that allokairic effectors can provide receptor-specific modulation of signaling by accessing the sequence divergent G protein binding interface on the receptor.

Collectively, our findings provide a framework for investigating transient events in GPCR signaling, with broad implications for targeting interactions and transitions to tune responses. We delineate receptor state transitions as transformations catalyzed both by cognate G proteins and allokairic effectors that bind to the ligand-bound receptors. We show that the qualitative effect of such effectors varies temporally, with a positive transient effect and inhibitory long-term effect (Supplementary Fig. 9b). Such non-intuitive behavior ‘emerges’ from a system for which the properties of each individual interaction is not sufficient to explain the observation of the whole^33^. This layer of complexity is dependent on the nature and concentration of effectors, which combinatorically increases the repertoire of options available to fine-tune GPCR activation states. The emergent behavior shown in our transient kinetic model fundamentally changes how we understand GPCR-G protein interactions, uncovering additional mechanisms to investigate GPCR signaling and regulation.

## Methods

### Reagents

Ascorbic acid, (−)-Isoproterenol (+)-bitartrate salt and clenbuterol hydrochloride were purchased from MilliporeSigma. Polyethyleneimine (PEI) 25 kDa linear polymer was purchased from PolySciences, Streptavidin-coated magnetic beads (Dynabeads) were purchased from New England Biolabs. Ni-NTA agarose was purchased from Qiagen. Synthetic peptides of the C-terminal α5 helix of the Gα subunit were synthesized at >95% purity from Genscript. Lyophilized peptides were dissolved in water and the concentration determined by the mass of the lyophilized peptide. Biotinylated Spep was synthesized at >95% purity from Genscript with an N-terminal biotin conjugation.

### Constructs

All constructs were expressed in pcDNA5/FRT (ThermoFisher). Construction of β2AR-mCerulean and β2AR SPASM sensor constructs were described previously^13,18,19^. SPASM senor constructs used here contain β2AR, mCitrine (FRET acceptor), 10 nM ER/K α-helix, mCerulean (FRET donor) and a 27 amino acid Gα C-terminal peptide. His-FLAG-SNAP-Nb6B9 in pBiEx was constructed by fusing Nb6B9 to the C-terminus of a SNAP tag with two glycine-serine-glycine repeats to allow for flexibility and free rotation between the domains.

### Cell culture and transfections

HEK293T-Flp-In (HEK293T, ThermoFisher, catalog number R78007) cells were cultured in Dulbecco’s Modified Eagle Media (DMEM) with 4.5 g/L D-glucose, and 10% Fetal Bovine Serum (FBS), 1% L-glutamine and 20 mM HEPES, pH 7.5. Cells were maintained at 5% humidity at 37 °C. For each membrane preparation, a 15 cm plate was transiently transfected using an optimized protocol with polyethylenimine (PEI, molecular weight 25,000, PolySciences). For a 15 cm plate, 10 μg of the sensor DNA was mixed with 40 μL PEI in 1 mL Opti-minimal essential medium media (ThermoFisher) and incubated for 15 min at 25 °C before adding to the cells. Media containing the transfection reagent was exchanged for fresh media after 4 h. Expression times for the tested constructs are as follows: β2AR-mCerulean (20 h), β2AR SPASM-Spep (22–26 h) and β2AR SPASM-R389ASpep (22–24 h). Expression and transfection efficiency was monitored with 20x and 40x magnification on a Nikon TS100 microscope equipped with a 100 W Hg-arc lamp and fluorescence filter cubes.

### Membrane preparations

Membranes were prepared as previously published^34^ from transiently transfected HEK293T cells expressing the desired receptor construct after transient transfection. Cells were harvested and collected in tissue culture media and washed once PBS (300 x g, 3 min, room temperature). Pelleted cells were resuspended in 8 mL of hypotonic buffer (10 mM HEPES, 50 mM EDTA, pH 7.4) with 1.5 μg/μL aprotonin, 1.5 μg/μL leupeptin and 1 mM DTT for 30 minutes. 5 μg/μL PMSF was added to the cell suspension, which was gently lysed in a chilled dounce homogenizer. Cell debris and nuclei were pelleted at 1000 x g for 5 min at 4 C and membranes pelleted from the supernatant in a TLA 100.4 rotor (135,000 x g for 25 min at 4 °C). Native membranes were washed in FRET buffer (20 mM HEPES pH 7.4, 5 mM KCl, 145 mM NaCl, 2 mM CaCl_2_ and 1 mM MgCl_2_) with 1.5 μg/μL aprotonin, 1.5 μg/μL leupeptin, 1 mM DTT and 5 μg/μL PMSF. Membranes were flash frozen and stored at −80 °C in FRET buffer with 12.5% sucrose.

### Nb6B9 purification

pBiEx vector containing His-Flag-SNAP-Nb6B9 was transformed into JM109(DE3) *E. coli* cells. Single colonies were grown overnight at 37 °C with shaking in Luria broth + carbenicillin. 500 mL terrific broth broth with carbenicillin was inoculated with 50 mL of the overnight culture and grown at 37 °C with shaking. Growth was monitored by measuring OD_600_ and the culture induced with 1 mM IPTG when OD_600_ = 1.0–1.2. The culture was moved to 18 °C for expression overnight (~16 h), and the cells pelleted in at 3000 x g for 15 minutes. The pellet was either stored at −80 °C or resuspended in lysis buffer (20 mM HEPES, pH 7.4, 400 mM NaCl, 10 mM imidazole, 5% glycerol, 0.1 mM DTT). The cell suspension was sonicated 10 s on/30 s off on ice until the cells were clearly lysed (total 5 min on). Cell debris was removed by centrifugation (14,000 x g for 30 min at 4 °C). Meanwhile, 6 mL Ni-NTA resin slurry (3 mL column volume) was washed 3x with lysis buffer for batch purification. The supernatant was added to the washed Ni-NTA resin and incubated for 1 h with rotation at 4 °C. Resin was pelleted with centrifugation (1000 x g for 2 min) and washed 3x with lysis buffer before elution with 400 mM imidazole. Further purification over a HiLoad S200 gel filtration column in size exclusion buffer (20 mM HEPES pH 7.4, 400 mM NaCl) isolated His-Flag-SNAP-Nb6B9. Fractions were pooled, concentrated and flash frozen for storage at −80 °C. Molecular weight and purity was confirmed using SDS-PAGE gel.

### Stopped flow kinetics

Stopped flow experiments were performed using a KinTek stopped flow attachment on a Fluoromax-4 flourometer (Horiba Scientific). The syringes and driver platform connect to an umbical leading to the cuvette, where mixing occurs. Fluorescent readings were measured in kinetic mode, with the excitation set to 430 nm and emission set to 475 nm (FRET donor, mCerulean) or 525 nm (FRET acceptor, mCitrine). To ensure proper quenching reactions and data analysis, the quenching reaction between N-bromo-succimide and N-acetyl-tryptophamide was measured^35^ (Supplementary Fig. 6). Data analysis and curve fitting was performed using MATLAB (R2020A, MathWorks). Each decay curve was an average of at least 5 injections.

The Nb6B9 quenching of β2AR-Spep SPASM FRET experiments were completed with 100 μM isoproterenol (iso) in all syringes. The system was flushed 3 times with millipure water, and 3 times with FRET buffer. Driver syringes were loaded with sample and the system primed with 500 μL of sample from each syringe. Syringe 1 contained FRET buffer, 100 μM iso and β2AR-Spep SPASM sensor (2 × 10^6 counts mCer). Syringe 2 contained FRET buffer + 100 μM iso with or without 20 μM Nb6B9. Each injection into the cuvette contained 20 μL from each syringe, for a total volume of 40 μL at a flow rate of 8 mL/sec. Kinetic readings were measured for a total of 2 min with an integration time of 4 ms and readings every 4 ms. Each reading was measured with an excitation of 430 nm (bandpass 10 nm) and an emission of either 475 nm or 525 nm (bandpass 10 nm). The fluorimeter reading was started at time *t* = 0 s with the KinTek injection at time *t* = 2 s. 5–10 injections were measured for each emission wavelength for the conditions with and without Nb6B9. Individual decay traces were analyzed to determine the start of each decay curve. The decays for each condition were averaged starting at the specified start position and continuing for 115 seconds. For each time point, the FRET ratio was determining by dividing the average 525 nm by the average 475 nm to give the FRET ratio over time.

The FRET ratio for iso alone and iso + Nb6B9 were fit for each membrane (Supplementary Fig. 7). First, the iso alone decay curve was fit to a single exponential decay using the custom equation $y={{ae}}^{-{bx}}+c$ on the MATLAB curve fitting tool (Curve Fitting Toolbox, MathWorks). The values of a (the range of the decay) and b were used to account for the decrease in FRET that occurred in the system in the absence of Nb6B9. Then, the iso + Nb6B9 curve was fit to a triple exponential decay using the custom equation $y={{ae}}^{-{bx}}+c{e}^{-{dx}}+f{e}^{-{gx}}+h$, where the values of a and b were constrained to match the values of the iso alone decay. The rates of the Nb6B9 decays, d and g, were averaged for each of the membranes in order to determine the average rates for the two states of the receptor-G protein interaction.

### β2AR affinity sequestration assays

Native membranes from HEK293T cells transiently transfected with β2AR-mCerulean were resuspended in FRET buffer with 0.1 mg/mL BSA, 100 μM iso and supplemented with 100 μM ascorbic acid were prepared. The concentration of β2AR-mCer was 1 × 10^6 counts mCerulean. Peptide solutions contained either no peptide, 10 μM Qpep, 30 μM Bio-Spep, or 30 μM bio-Spep with 10 μM Qpep. Dynabeads were washed with FRET buffer + 0.1 mg/mL BSA twice and resuspended in FRET buffer + 0.1 mg/mL BSA. 50 μL of peptide solutions were added to 1.5 mL tubes. At time *t* = 0, 250 uL of the β2AR-mCer was added to each tube and allow to incubate at room temperature. At time *t* = 5 min, 100 μL of the solution was placed into a clean tube to be measured as the input control and 40 μL Dynabeads was added to the remaining 200 μL in the tube and incubated at room temperature for 5 minutes. After 5 min, the Dynabeads were sequestered using a Neodymium disc magnet N52 (20 × 40 mm). After all the Dynabeads were sequestered, the supernatant was removed and placed in a clean tube to measure the counts of mCerulean. The fraction of β2AR-mCer in supernatant was calculated by dividing the background subtracted mCerulean counts for the bound samples by the background subtracted input control. Background spectra were taken from membranes prepared from untransfected Hek293Tcells. Fraction bound = 1- fraction in supernatant.

### FRET assays

Native membranes from HEK293T cells transiently transfected with β2AR-Spep-SPASM or β2AR-R389ASpep-SPASM sensor were resuspended in FRET buffer to 1*10^6 counts mCerulean. The membranes were sonicated for 15 s on ice to homogenize the solution. In assays with soluble peptides, the desired concentration of the peptide was added after sonication. Then, 90 μL of membrane solution was aliquoted into 10 tubes; 5 tubes received 100 μM agonist (isoproterenol, iso or clenbuterol, clen) treatment and 5 tubes received buffer. Agonist solutions contained 1 mM in FRET buffer with 1 mM ascorbic acid and buffer solutions contained FRET buffer with 1 mM ascorbic acid. Samples were stimulated for 5 min with shaking (300 rpm) at 25 °C with 10 μL of either agonist or buffer solutions. After 5 min of stimulation, 90 μL of the solution was transferred to an optical quartz cuvette (3-3.0-SOG-3, Starna Cells, Inc) and the spectra was measured in a Fluormax-4 fluorometer (Horiba Scientific). Sample was excited at 430 nm (bandpass 8 nm) and a scanning emission from 450 to 600 nm (bandpass 4 nm) was measured. The typical FRET spectrum has a peak at 475 nm (mCerulean, donor) and at 525 nm (mCitrine, acceptor). After background correction, the FRET ratio of 525 nm emission to 475 nm emission was determined for agonist and buffer. To determine the change in FRET (ΔFRET), the average FRET ratio of the buffer conditions was subtracted from the average of the agonist conditions.

### Mathematical model

We formulated a mathematical model based on the reactions shown in Supplementary Fig 5a. All reaction rates were assumed to follow mass-action kinetics. A mass balance on each chemical species results in an ordinary differential equation that equates the rate of change of species concentration to the net rate resulting from reactions that result in its formation and reactions where that species is a reactant. These equations are listed in Supplementary Fig 5b. The resulting steady-state concentrations were numerically calculated using the parameters defined in Table S1 by integrating until the concentration change is below the specified tolerance. Parameters listed in Table S1 are in the range of parameters reported for similar association/dissociation reactions reported in the Bionumbers database^36^. All calculations were carried out using MATLAB R2020a (MathWorks). For transient simulations, the integration was carried out for a time corresponding to the time for the experimental measurement.

### Statistics and reproducibility

For each experiment, a single n was performed with either one passage of cell or membrane preparation derived from a single batch of cells. All experiments were repeated at least 3 times (*n* = 3). ΔFRET measurements on a single membrane preparation (*n* = 1), were repeated 4 times (average of 4 FRET measurements of the agonist – 4 FRET measurements of buffer). Stopped flow decay curves show the decrease in FRET (525 nm/475 nm) over time. To measure the FRET on a single membrane preparation, the counts at 525 nm or 475 nm from at least 5 injections were averaged. One cAMP experiment is the average of 4 measurements for a given condition. Statistics were performed as described in figure legends, using t-tests or 2-way ANOVA with multiple comparisons, where appropriate, in Prism 9.

### Reporting summary

Further information on research design is available in the Nature Research Reporting Summary linked to this article.

## Supplementary information

Supplementary Information

Reporting Summary

## Data Availability

The data generated in this study is available in the Source Data File provided with this paper. Bionumbers database was used to confirm computational parameters (ID: 103809) Source data are provided with this paper.

## Source data

Source Data

## Code availability

The detailed equations and parameters that are the basis for the Matlab code are provided in the supplementary information. The Matlab files will be made available upon request to the corresponding authors.
