# Supplementary material for: Kinetic model of GPCR-G protein interactions reveals allokairic modulation of signaling
Source: Nat Commun. 2022 Mar 8;13:1202. doi: 10.1038/s41467-022-28789-5 (PMC8904551; doi:10.1038/s41467-022-28789-5)
Supplement: Supplementary file 1 — Supplementary Information [file 41467_2022_28789_MOESM1_ESM.pdf]

Supplementary Information for

Kinetic model of GPCR-G protein interactions reveals allokairic modulation of signaling

Kelly J. Culhane[1], Tejas M. Gupte[1], Indrani Madhugiri[2], Chetan J. Gadgil[2,3]*, Sivaraj Sivaramakrishnan[1]*

Correspondence to: sivaraj@umn.edu; cj.gadgil@ncl.res.in

This PDF file includes:

Supplementary Note
Supplemental Figures 1-10
Supplemental Tables 1 & 2

Supplementary Note 1

Proof: Addition of E_{Ak} does not increase steady-state level of bound G protein

Consider reaction scheme and equations in Fig S5. Assuming that the principle of microscopic reversibility is applicable, each reversible reaction is individually at equilibrium, leading to the algebraic equations below, where concentrations represent equilibrium values.

$$k_{onHR'E}\,[HR']\,[E_{Ak}] = k_{offHR'E}\,[HR'E_{Ak}]$$
$$k_{forwardE}\,[HR'E_{Ak}] = k_{reverseE}\,[HR^*E_{Ak}]$$
$$k_{onHR^*E}\,[HR^*]\,[E_{Ak}] = k_{offHR^*E}\,[HR^*E_{Ak}]$$
$$k_{forward}\,[HR'] = k_{reverse}\,[HR^*]$$
$$k_{onHR'G}\,[HR']\,[G] = k_{offHR'G}\,[HR'G]$$
$$k_{forwardG}\,[HR'G] = k_{reverseG}\,[HR^*G]$$
$$k_{onHR^*G}\,[HR^*]\,[G] = k_{offHR^*G}\,[HR^*G]$$

This can be restated as

$$\frac{[HR']}{[HR^*]} = \frac{k_{reverse}}{k_{forward}} = \frac{k_{reverseE}}{k_{forwardE}} \cdot \frac{K_{HR'E_{Ak}}}{K_{HR^*E_{Ak}}} = \frac{k_{reverseG}}{k_{forwardG}} \cdot \frac{K_{HR'G}}{K_{HR^*G}}$$

or

$$\frac{[HR']}{[HR^*]} = K_{act} = K_1 \cdot \frac{K_{HR'E_{Ak}}}{K_{HR^*E_{Ak}}} = K_2 \cdot \frac{K_{HR'G}}{K_{HR^*G}} \qquad (1)$$

where: $K_{HR'E_{Ak}} = K_E = \frac{k_{offHR'E}}{k_{onHR'E}}$, $K_{HR^*E_{Ak}} = \frac{k_{offHR^*E}}{k_{onHR^*E}}$, $K_1 = \frac{k_{reverseE}}{k_{forwardE}}$, $K_{act} = \frac{k_{reverse}}{k_{forward}}$, $K_{HR'G} = K_D = \frac{k_{offHR'G}}{k_{onHR'G}}$, $K_{HR^*G} = \frac{K_D}{\alpha} = \frac{k_{offHR^*G}}{k_{onHR^*G}}$ and $K_2 = \alpha\, K_{act} = \frac{k_{reverseG}}{k_{forwardG}}$

Let $E_{Ak}B$ and GB denote bound forms of E_{Ak} and G respectively:

$$[E_{Ak}B] = [HR'E_{Ak}] + [HR^*E_{Ak}] = (1 + K_1) \cdot [HR^*E_{Ak}] \qquad (2)$$

$$[GB] = [HR'G] + [HR^*G] = (1 + K_2) \cdot [HR^*G] \qquad (3)$$

Since total HR, E_{Ak} and G is constant, we get the conservation equations:

$$E_{Aktot} = [E_{Ak}] + [E_{Ak}B] \qquad (4)$$

$$G_{tot} = [G] + [GB] \qquad (5)$$

$$HR'_{tot} = [HR'] + [HR^*] + [E_{Ak}B] + [GB] \qquad (6)$$

At steady state, setting derivative of the differential equations in Fig S5B to zero, we get:

$$[E_{Ak}] = \left(\frac{K_{HR^*E_{Ak}}}{1 + K_1}\right) \cdot \frac{[E_{Ak}B]}{[HR^*]} \qquad (7)$$

$$[G] = \left(\frac{K_{HR^*G}}{1 + K_2}\right) \cdot \frac{[GB]}{[HR^*]} \qquad (8)$$

From (4) and (7)

$$E_{Aktot} = [E_{Ak}B]\left(1 + \left(\frac{K_{HR^*E_{Ak}}}{1 + K_1}\right)\frac{1}{[HR^*]}\right) \qquad (9)$$

From (5) and (8),

$$G_{tot} = [GB]\left(1 + \left(\frac{K_{HR^*G}}{1+K_2}\right)\frac{1}{[HR^*]}\right) \tag{10}$$

Substituting in (6), we get

$$HR'_{tot} = (1 + \ k_{act})[HR^*] + [E_{Ak}B] + [GB] \tag{11}$$

$$HR'_{tot} = [HR^*]\left(1 + \ k_{act} + \frac{E_{Aktot}}{[HR^*] + \left(\frac{K_{HR^*E_{Ak}}}{1+K_1}\right)} + \frac{G_{tot}}{[HR^*] + \left(\frac{K_{HR^*G}}{1+K_2}\right)}\right) \tag{12}$$

Taking partial derivative of (10) with respect to E_{Aktot}:

$$0 = \frac{\partial[GB]}{\partial E_{Aktot}}\left(1 + \frac{K_{HR^*G}}{(1+K_2)[HR^*]}\right) + [GB]\left(\frac{-K_{HR^*G}}{(1+K_2)[HR^*]^2}\cdot\frac{\partial[HR^*]}{\partial E_{Aktot}}\right)$$

$$\frac{\partial[HR^*]}{\partial E_{Aktot}} = \left(\frac{a_{11}}{a_{12}}\right)\frac{\partial[GB]}{\partial E_{Aktot}} \tag{13}$$

where: $a_{11} = 1 + \left(\frac{K_{HR^*G}}{(1+K_2)[HR^*]}\right)$ and $a_{12} = \left(\frac{K_{HR^*G}}{1+K_2}\right)\frac{[GB]}{[HR^*]^2}$

Taking partial derivative of (11) with respect to E_{Aktot}, and substituting using (13),

$$0 = \frac{\partial[HR^*]}{\partial E_{Aktot}}(1 + \ k_{act}) + \frac{\partial[E_{Ak}B]}{\partial E_{Ak_{tot}}} + \frac{\partial[GB]}{\partial E_{Aktot}}$$

$$\frac{\partial[E_{Ak}B]}{\partial E_{Aktot}} = -a_{21}\cdot\frac{\partial[GB]}{\partial E_{Aktot}} \text{ where } a_{21} = 1 + \frac{a_{11}}{a_{12}}(1 + \ k_{act}) \tag{14}$$

Taking partial derivative of (9) with respect to E_{Aktot}, and substituting using (13) and (14),

$$1 = \frac{\partial[E_{Ak}B]}{\partial E_{Aktot}}\left(1 + \frac{K_{HR^*E_{Ak}}}{(1+K_1)[HR^*]}\right) + [E_{Ak}B]\left(\frac{-K_{HR^*E_{Ak}}}{(1+K_1)[HR^*]^2}\cdot\frac{\partial[HR^*]}{\partial E_{Aktot}}\right)$$

$$1 = -\left(a_{21}\left(1 + \frac{K_{HR^*E_{Ak}}}{(1+K_1)[HR^*]}\right) + \left(\frac{a_{11}}{a_{12}}\cdot\frac{K_{HR^*E_{Ak}}}{1+K_1}\cdot\frac{[E_{Ak}B]}{[HR^*]^2}\right)\right)\frac{\partial[GB]}{\partial E_{Aktot}}$$

$$\frac{\partial[GB]}{\partial E_{Aktot}} = \frac{-1}{a_{31}} \tag{15}$$

where: $a_{31} = a_{21}\left(1 + \frac{K_{HR^*E_{Ak}}}{(1+K_1)[HR^*]}\right) + \left(\frac{a_{11}}{a_{12}}\right)\left(\frac{K_{HR^*E_{Ak}}}{1+K_1}\right)\left(\frac{[E_{Ak}B]}{[HR^*]^2}\right)$

Since a_{31} is non-negative, $\frac{\partial[GB]}{\partial E_{Aktot}} < 0$, proving that steady state bound G-protein levels cannot increase when a competing modulator is added, irrespective of the binding affinities.

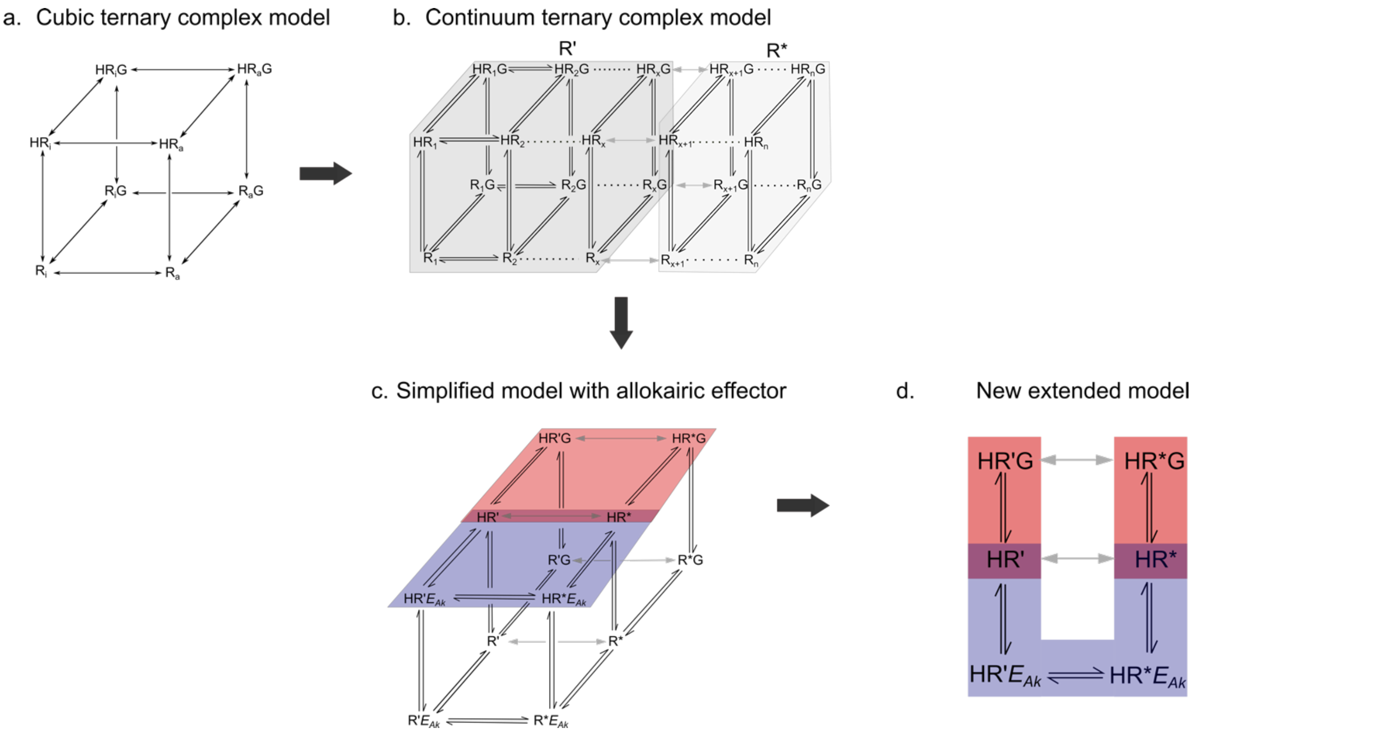

Supplemental Figure 1: Continuum model of GPCR signaling. (a) Cubic ternary complex model[3-5] describes hormone (H) and G protein binding to receptor active (R_a) and inactive (R_i) states **(b)** New continuum ternary complex model shows hormone and G protein binding to progressively more active receptor states (R_n). A rate limiting conformational change occurs between states $R_x \rightarrow R_{x+1}$. Lumping receptor states before (R') and after (R*) this rate limiting step leads to a simplified model **(c)** Simplified model showing additional interaction between R' and R* with an allokairic effector (E_{Ak}). Hormone bound receptor (HR', HR*) interactions with E_{Ak} and G protein are shown in blue and red, respectively. **(d)** new extended model shows interactions of E_{Ak} and G protein during ternary complex formation.

Supplemental Figure 2: Prism Tesseract Model of ligand-receptor-G protein engagement. The model captures the inclusions of multiple hormones, receptors, and G proteins/modulators. H, R and G in the prism tesseract model below represent the ith receptor, jth G protein/modulator, and the kth hormone.

Multiple ligands, multiple receptors, and multiple G-proteins; each can have two distinct states

H: Hormone (ligand L)

R: Receptor

G: G-protein (if G_j is cognate G-protein, then $G_{j'}$, is noncognate (E_{Ak}))

$\boldsymbol{H_k R_i^x G_j^y}$

Superscript denotes species state, and subscript denotes species identity

Hormone H can exist in bound and unbound forms	k: hormone identity = {1, 2, ………., n_H}
x: receptor state = {R‘, R*}	i: receptor identity = {1, 2, ………., n_R}
y: G-protein state = {$G^{inactive}$, G^{active}}	j: G-protein identity = {1, 2, ………., n_G}

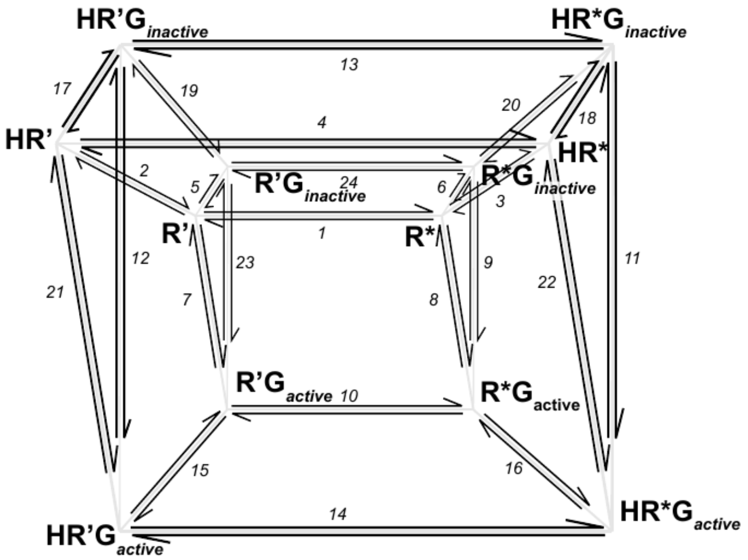

The unbound hormone H (ligand L), and the two distinct states of unbound G protein ($G^{inactive}$ and G^{active}) are not explicitly shown in the above tesseract figure. Also, the figure does not explicitly depict the spontaneous (uncatalyzed) transition between the two G protein states.

Reactions:

0: $G_j^{inactive} \leftrightarrow G_j^{active}$

1: $R_i' \leftrightarrow R_i^*$

2: $H_k + R_i' \leftrightarrow H_k R_i'$

3: $H_k + R_i^* \leftrightarrow H_k R_i^*$

4: $H_k R_i' \leftrightarrow H_k R_i^*$

5: $R_i' + G_j^{inactive} \leftrightarrow R_i' G_j^{inactive}$

6: $R_i^* + G_j^{inactive} \leftrightarrow R_i^* G_j^{inactive}$

7: $R_i' + G_j^{active} \leftrightarrow R_i' G_j^{active}$

8: $R_i^* + G_j^{active} \leftrightarrow R_i^* G_j^{active}$

9: $R_i^* G_j^{inactive} \leftrightarrow R_i^* G_j^{active}$

10: $R_i' G_j^{active} \leftrightarrow R_i^* G_j^{active}$

11: $H_k R_i^* G_j^{inactive} \leftrightarrow H_k R_i^* G_j^{active}$

12: $H_k R_i' G_j^{inactive} \leftrightarrow H_k R_i' G_j^{active}$

13: $H_k R_i' G_j^{inactive} \leftrightarrow H_k R_i^* G_j^{inactive}$

14: $H_k R_i' G_j^{active} \leftrightarrow H_k R_i^* G_j^{active}$

15: $H_k + R_i' G_j^{active} \leftrightarrow H_k R_i' G_j^{active}$

16: $H_k + R_i^* G_j^{active} \leftrightarrow H_k R_i^* G_j^{active}$

17: $H_k R_i' + G_j^{inactive} \leftrightarrow H_k R_i' G_j^{inactive}$

18: $H_k R_i^* + G_j^{inactive} \leftrightarrow H_k R_i^* G_j^{inactive}$

19: $H_k + R_i' G_j^{inactive} \leftrightarrow H_k R_i' G_j^{inactive}$

20: $H_k + R_i^* G_j^{inactive} \leftrightarrow H_k R_i^* G_j^{inactive}$

21: $H_k R_i' + G_j^{active} \leftrightarrow H_k R_i' G_j^{active}$

22: $H_k R_i^* + G_j^{active} \leftrightarrow H_k R_i^* G_j^{active}$

23: $R_i' G_j^{inactive} \leftrightarrow R_i' G_j^{active}$

24: $R_i' G_j^{inactive} \leftrightarrow R_i^* G_j^{inactive}$

Supplemental Figure 3: Tesseract model equations.

$$\frac{d[H_k]}{dt} = \sum_{i=1}^{n_R}\sum_{j=1}^{n_G}\Big(-k2^+_{ik}[H_k][R'_i] + k2^-_{ik}[H_kR'_i] - k3^+_{ik}[H_k][R^*_i] + k3^-_{ik}[H_kR^*_i] - k15^+_{ijk}[H_k][R'_iG_j^{active}] + k15^-_{ijk}[H_kR'_iG_j^{active}] \\ - k16^+_{ijk}[H_k][R^*_iG_j^{active}] + k16^-_{ijk}[H_kR^*_iG_j^{active}] - k19^+_{ijk}[H_k][R'_iG_j^{inactive}] + k19^-_{ijk}[H_kR'_iG_j^{inactive}] \\ - k20^+_{ijk}[H_k][R^*_iG_j^{inactive}] + k20^-_{ijk}[H_kR^*_iG_j^{inactive}]\Big)$$

$$\frac{d[R'_i]}{dt} = k1^-_i[R^*_i] - k1^+_i[R'_i] + \sum_{k=1}^{n_H}\left(k2^-_{ik}[H_kR'_i] - k2^+_{ik}[H_k][R'_i]\right) + \sum_{j=1}^{n_G}\begin{pmatrix} k5^-_{ij}[R'_iG_j^{inactive}] - k5^+_{ij}[R'_i][G_j^{inactive}] \\ +k7^-_{ij}[R'_iG_j^{active}] - k7^+_{ij}[R'_i][G_j^{active}] \end{pmatrix}$$

$$\frac{d[R^*_i]}{dt} = k1^+_i[R'_i] - k1^-_i[R^*_i] + \sum_{k=1}^{n_H}\left(k3^-_{ik}[H_kR^*_i] - k3^+_{ik}[H_k][R^*_i]\right) + \sum_{j=1}^{n_G}\begin{pmatrix} k6^-_{ij}[R^*_iG_j^{inactive}] - k6^+_{ij}[R^*_i][G_j^{inactive}] \\ +k8^-_{ij}[R^*_iG_j^{active}] - k8^+_{ij}[R^*_i][G_j^{active}] \end{pmatrix}$$

$$\frac{d[G_j^{active}]}{dt} = k0^+_j[G_j^{inactive}] - k0^-_j[G_j^{active}] + \sum_{k=1}^{n_H}\sum_{i=1}^{n_R}\begin{pmatrix} k7^-_{ij}[R'_iG_j^{active}] - k7^+_{ij}[R'_i][G_j^{active}] \\ +k8^-_{ij}[R^*_iG_j^{active}] - k8^+_{ij}[R^*_i][G_j^{active}] \\ +k21^-_{ijk}[H_kR'_iG_j^{active}] - k21^+_{ijk}[H_kR'_i][G_j^{active}] \\ +k22^-_{ijk}[H_kR^*_iG_j^{active}] - k22^+_{ijk}[H_kR^*_i][G_j^{active}] \end{pmatrix}$$

$$\frac{d[G_j^{inactive}]}{dt} = k0^-_j[G_j^{active}] - k0^+_j[G_j^{inactive}] + \sum_{k=1}^{n_H}\sum_{i=1}^{n_R}\begin{pmatrix} k5^-_{ij}[R'_iG_j^{inactive}] - k5^+_{ij}[R'_i][G_j^{inactive}] \\ +k6^-_{ij}[R^*_iG_j^{inactive}] - k6^+_{ij}[R^*_i][G_j^{inactive}] \\ +k17^-_{ijk}[H_kR'_iG_j^{inactive}] - k17^+_{ijk}[H_kR'_i][G_j^{inactive}] \\ +k18^-_{ijk}[H_kR^*_iG_j^{inactive}] - k18^+_{ijk}[H_kR^*_i][G_j^{inactive}] \end{pmatrix}$$

$$\frac{d[H_kR'_i]}{dt} = k2^+_{ik}[H_k][R'_i] - (k2^-_{ik} + k4^+_{ik})[H_kR'_i] + k4^-_{ik}[H_kR^*_i] + \sum_{j=1}^{n_G}\begin{pmatrix} k17^-_{ijk}[H_kR'_iG_j^{inactive}] - k17^+_{ijk}[H_kR'_i][G_j^{inactive}] \\ +k21^-_{ijk}[H_kR'_iG_j^{active}] - k21^+_{ijk}[H_kR'_i][G_j^{active}] \end{pmatrix}$$

$$\frac{d[H_kR^*_i]}{dt} = k3^+_{ik}[H_k][R^*_i] - (k3^-_{ik} + k4^-_{ik})[H_kR^*_i] + k4^+_{ik}[H_kR'_i] + \sum_{j=1}^{n_G}\begin{pmatrix} k18^-_{ijk}[H_kR^*_iG_j^{inactive}] - k18^+_{ijk}[H_kR^*_i][G_j^{inactive}] \\ +k22^-_{ijk}[H_kR^*_iG_j^{active}] - k22^+_{ijk}[H_kR^*_i][G_j^{active}] \end{pmatrix}$$

$$\frac{d[R'_iG_j^{inactive}]}{dt} = k5^+_{ij}[R'_i][G_j^{inactive}] + k23^-_{ij}[R'_iG_j^{active}] \\ -(k5^-_{ij} + k23^+_{ij} + k24^+_{ij})[R'_iG_j^{inactive}] + k24^-_{ij}[R^*_iG_j^{inactive}] \\ + \sum_{k=1}^{n_H}\left(k19^-_{ijk}[H_kR'_iG_j^{inactive}] - k19^+_{ijk}[H_k][R'_iG_j^{inactive}]\right)$$

$$\frac{d[R^*_iG_j^{inactive}]}{dt} = k6^+_{ij}[R^*_i][G_j^{inactive}] + k9^-_{ij}[R^*_iG_j^{active}] \\ -(k6^-_{ij} + k9^+_{ij} + k24^-_{ij})[R^*_iG_j^{inactive}] + k24^+_{ij}[R'_iG_j^{inactive}] \\ + \sum_{k=1}^{n_H}\left(k20^-_{ijk}[H_kR^*_iG_j^{inactive}] - k20^+_{ijk}[H_k][R^*_iG_j^{inactive}]\right)$$

$$\frac{d[R'_iG_j^{active}]}{dt} = k7^+_{ij}[R'_i][G_j^{active}] + k23^+_{ij}[R'_iG_j^{inactive}] \\ -(k7^-_{ij} + k23^-_{ij} + k10^+_{ij})[R'_iG_j^{active}] + k10^-_{ij}[R^*_iG_j^{active}] \\ + \sum_{k=1}^{n_H}\left(k15^-_{ijk}[H_kR'_iG_j^{active}] - k15^+_{ijk}[H_k][R'_iG_j^{active}]\right)$$

$$\frac{d[R^*_iG_j^{active}]}{dt} = k8^+_{ij}[R^*_i][G_j^{active}] + k9^+_{ij}[R^*_iG_j^{inactive}] \\ -(k8^-_{ij} + k9^-_{ij} + k10^-_{ij})[R^*_iG_j^{active}] + k10^+_{ij}[R'_iG_j^{active}] \\ + \sum_{k=1}^{n_H}\left(k16^-_{ijk}[H_kR^*_iG_j^{active}] - k16^+_{ijk}[H_k][R^*_iG_j^{active}]\right)$$

$$\frac{d[H_kR'_iG_j^{active}]}{dt} = k14^-_{ijk}[H_kR^*_iG_j^{active}] - k14^+_{ijk}[H_kR'_iG_j^{active}] \\ -k12^-_{ijk}[H_kR'_iG_j^{active}] + k12^+_{ijk}[H_kR'_iG_j^{inactive}] \\ +k15^+_{ijk}[H_k][R'_iG_j^{active}] - k15^-_{ijk}[H_kR'_iG_j^{active}] \\ -k21^-_{ijk}[H_kR'_iG_j^{active}] + k21^+_{ijk}[H_kR'_i][G_j^{active}]$$

$$\frac{d[H_kR^*_iG_j^{active}]}{dt} = k14^+_{ijk}[H_kR'_iG_j^{active}] - k14^-_{ijk}[H_kR^*_iG_j^{active}] \\ -k11^-_{ijk}[H_kR^*_iG_j^{active}] + k11^+_{ijk}[H_kR^*_iG_j^{inactive}] \\ +k16^+_{ijk}[H_k][R^*_iG_j^{active}] - k16^-_{ijk}[H_kR^*_iG_j^{active}] \\ -k22^-_{ijk}[H_kR^*_iG_j^{active}] + k22^+_{ijk}[H_kR^*_i][G_j^{active}]$$

$$\frac{d[H_kR'_iG_j^{inactive}]}{dt} = k13^-_{ijk}[H_kR^*_iG_j^{inactive}] + k12^-_{ijk}[H_kR'_iG_j^{active}] \\ +k19^+_{ijk}[H_k][R'_iG_j^{inactive}] + k17^+_{ijk}[H_kR'_i][G_j^{inactive}] \\ -(k13^+_{ijk} + k12^+_{ijk} + k19^-_{ijk} + k17^-_{ijk})[H_kR'_iG_j^{inactive}]$$

$$\frac{d[H_kR^*_iG_j^{inactive}]}{dt} = k13^+_{ijk}[H_kR'_iG_j^{inactive}] + k11^-_{ijk}[H_kR^*_iG_j^{active}] \\ +k20^+_{ijk}[H_k][R^*_iG_j^{inactive}] + k18^+_{ijk}[H_kR^*_i][G_j^{inactive}] \\ -(k13^-_{ijk} + k11^+_{ijk} + k20^-_{ijk} + k18^-_{ijk})[H_kR^*_iG_j^{inactive}]$$

Allosteric binding of G proteins: If there are multiple modulators binding to R_i^x, say a cognate G_{j1}^y and noncognate G_{j2}^y, competitive modulators will replace G in the inner prism of the truncated tesseract; and noncompetitive mediators will in addition form $R_i^xG_{j1}^yG_{j2}^y$ (and $H_kR_i^xG_{j1}^yG_{j2}^y$) complexes. Allosteric modulators will lead to binding of G_{j2}^y to all nodes of this truncated tesseract. And the above equations will have to be modified accordingly to accommodate for change due to these additional reactions.

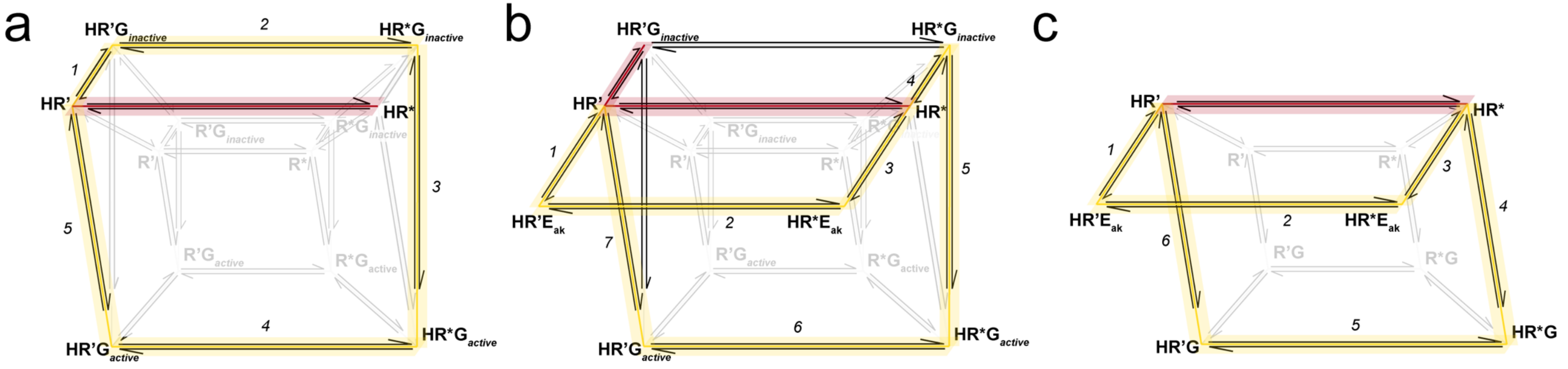

$G_{inactive} = G_{GDP},\ G_{empty}$

$G_{active} = G_{GTP}$

Supplemental Figure 4: Tesseract model extensions. (a) Sequence of events leading to G protein activation (Gregorio et al., Nature, 2017) highlighting the conformation change in the receptor triggered by the G protein (HR' to HR*). (b) Weak interaction between HR' and G protein (red arrow) is overcome by allokairic effector (E_{Ak}). (c) Simplified version of (b) with a single G protein interaction state. Numbering depicts the kinetically favorable sequence of events in each scheme.

a.

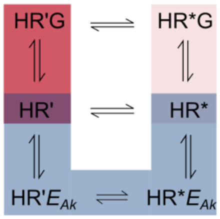

Receptor binding/interconversion equations

$$HR' + G \underset{k_{off\,HR'G}}{\overset{k_{on\,HR'G}}{\rightleftharpoons}} HR'G \qquad HR' + E_{Ak} \underset{k_{off\,HR'E}}{\overset{k_{on\,HR'E}}{\rightleftharpoons}} HR'E_{Ak}$$

$$HR^* + G \underset{k_{off\,HR^*G}}{\overset{k_{on\,HR^*G}}{\rightleftharpoons}} HR^*G \qquad HR^* + E_{Ak} \underset{k_{off\,HR^*E}}{\overset{k_{on\,HR^*E}}{\rightleftharpoons}} HR^*E_{Ak}$$

$$HR'G \underset{k_{reverse\,G}}{\overset{k_{forward\,G}}{\rightleftharpoons}} HR^*G \qquad HR'E_{Ak} \underset{k_{reverse\,E}}{\overset{k_{forward\,E}}{\rightleftharpoons}} HR^*E_{Ak}$$

$$HR' \underset{k_{reverse}}{\overset{k_{forward}}{\rightleftharpoons}} HR^*$$

Receptor binding/interconversion rates

Receptor interconversion rates		G protein binding rates		E_{Ak} binding rates	
$k_{forward}$	0.001 s^{-1}	$k_{on\,HR'G}$	0.01 $\mu M^{-1}s^{-1}$	$k_{on\,HR'E}$	0.1 $\mu M^{-1}s^{-1}$
$k_{reverse}$	0.01 s^{-1}	$k_{off\,HR'G}$	0.3 s^{-1}	$k_{off\,HR'E}$	3 s^{-1}
$k_{forward\,G}$	0.001 s^{-1}	$k_{on\,HR^*G}$	0.008 $\mu M^{-1}s^{-1}$	$k_{on\,HR^*E}$	0.1 $\mu M^{-1}s^{-1}$
$k_{reverse\,G}$	0.00025 s^{-1}	$k_{off\,HR^*G}$	0.006 s^{-1}	$k_{off\,HR^*E}$	3 s^{-1}
$k_{forward\,E}$	0.1 s^{-1}				
$k_{reverse\,E}$	1 s^{-1}				

b. Change in each species over time

$$dHR'/dt = -k_{on\,HR'G}[HR'][G] + k_{off\,HR'G}[HR'G] - k_{on\,HR'E}[HR'][E_{Ak}] + k_{off\,HR'E}[HR'E_{Ak}] - k_{forward}[HR'] + k_{reverse}[HR^*]$$

$$dHR^*/dt = -k_{on\,HR^*G}[HR^*][G] + k_{off\,HR^*G}[HR^*G] - k_{on\,HR^*E}[HR^*][E_{Ak}] + k_{off\,HR^*E}[HR^*E_{Ak}] - k_{reverse}[HR^*] + k_{forward}[HR']$$

$$dG/dt = -k_{on\,HR'G}[HR'][G] + k_{off\,HR'G}[HR'G] - k_{on\,HR^*G}[HR^*][G] + k_{off\,HR^*G}[HR^*G]$$

$$dE_{Ak}/dt = -k_{on\,HR'E}[HR'][E_{Ak}] + k_{off\,HR'E}[HR'E_{Ak}] - k_{on\,HR^*E}[HR^*][E_{Ak}] + k_{off\,HR^*E}[HR^*E_{Ak}]$$

$$dHR'G/dt = k_{on\,HR'G}[HR'][G] - k_{off\,HR'G}[HR'G] - k_{forward\,G}[HR'G] + k_{reverse\,G}[HR^*G]$$

$$dHR^*G/dt = k_{on\,HR^*G}[HR^*][G] - k_{off\,HR^*G}[HR^*G] - k_{reverse\,G}[HR^*G] + k_{forward\,G}[HR'G]$$

$$dHR'E_{Ak}/dt = k_{on\,HR'E}[HR'][E_{Ak}] - k_{off\,HR'E}[HR'E_{Ak}] - k_{forward\,E}[HR'E_{Ak}] + k_{reverse\,E}[HR^*E_{Ak}]$$

$$dHR^*E_{Ak}/dt = k_{on\,HR^*E}[HR^*][E_{Ak}] - k_{off\,HR^*E}[HR^*E_{Ak}] - k_{reverse\,E}[HR^*E_{Ak}] + k_{forward\,E}[HR'E_{Ak}]$$

c. Integration

All rates used for these simulations are listed in panel A
Integration time is 100,000 s
Transient (300 s) time indicated by vertical dashed line

Initial Conditions†	
$[HR']_{t=0}$	0.01 μM
$[G]_{t=0}$	10 μM
$[E_{Ak}]_{t=0}$	10 μM

†Unless specified, initial concentrations = 0

10 μM G protein + 0 μM E_{Ak}

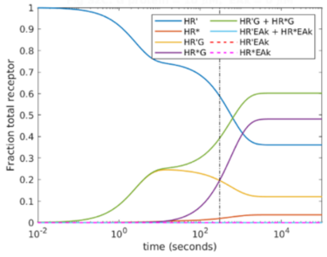

10 μM G protein + 10 μM E_{Ak}

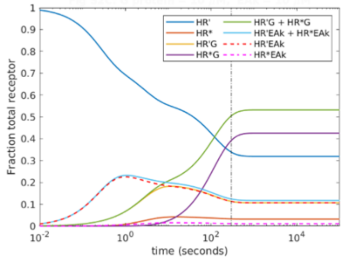

10 μM G protein + 30 μM E_{Ak}

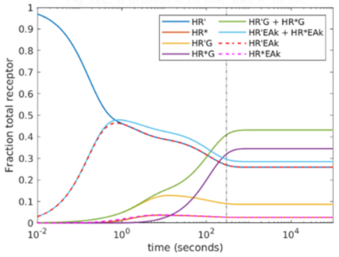

Supplemental Figure 5: Simulation parameters and single integration examples. (a) Binding and interconversion equations used in the simulations with parameters indicated in each equation and specified in the parameter table **(b)** Derivatives of the equations in panel A were used to determine the change of each species over time **(c)** Integration details and example 100,000 s integrations with 10 μM G protein with 0 μM E_{Ak}, 10 μM E_{Ak} or 30 μM E_{Ak}.

a. No peptide SPASM sensor FRET decay controls

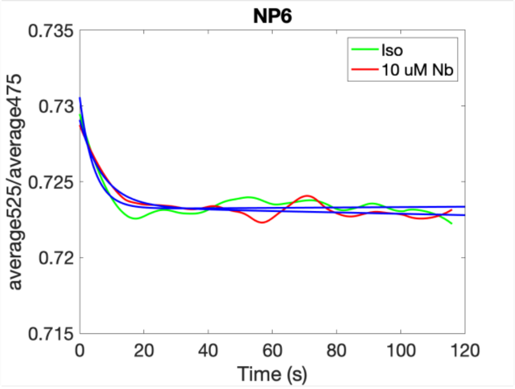

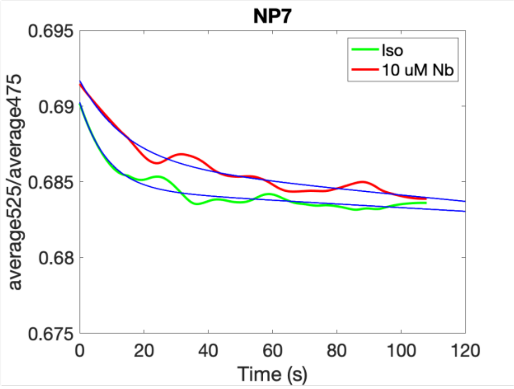

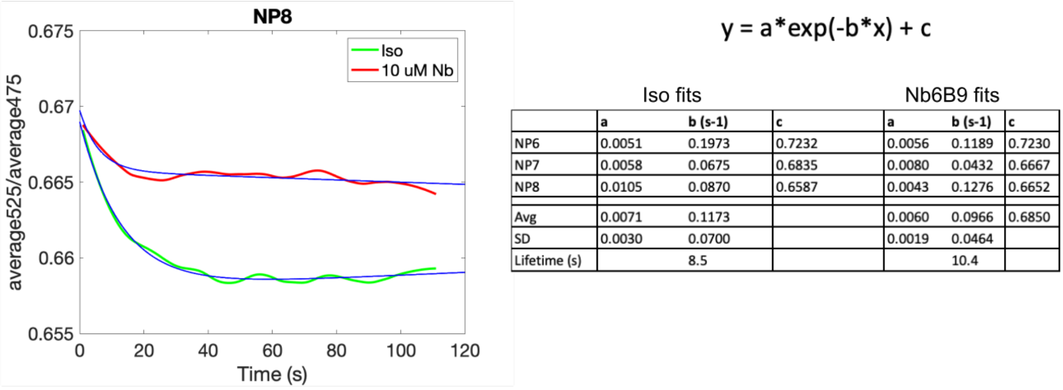

$$y = a*\exp(-b*x) + c$$

	Iso fits			Nb6B9 fits		
	a	b (s-1)	c	a	b (s-1)	c
NP6	0.0051	0.1973	0.7232	0.0056	0.1189	0.7230
NP7	0.0058	0.0675	0.6835	0.0080	0.0432	0.6667
NP8	0.0105	0.0870	0.6587	0.0043	0.1276	0.6652
Avg	0.0071	0.1173		0.0060	0.0966	0.6850
SD	0.0030	0.0700		0.0019	0.0464	
Lifetime (s)		8.5			10.4	

b. NBS quenching of NATA fluorescence control

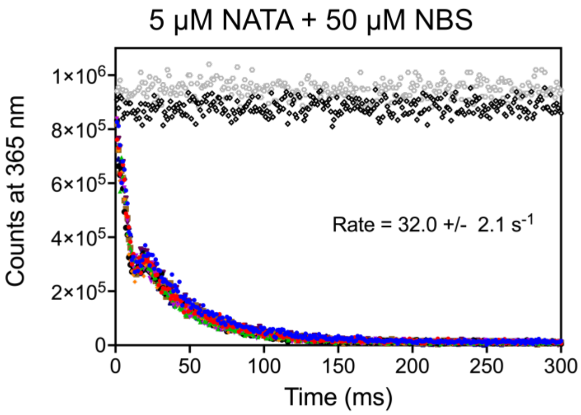

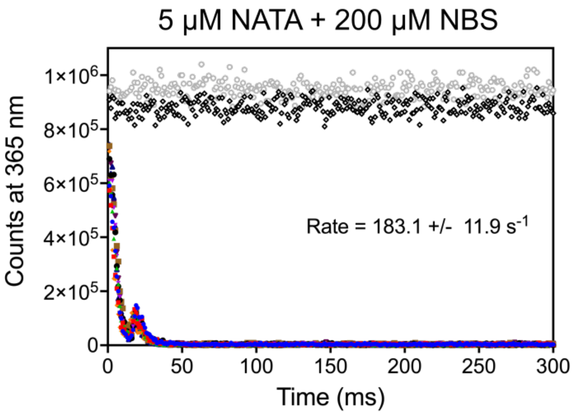

Supplemental Figure 6: Stopped flow controls and supporting data. (a) β2AR-No peptide SPASM sensors FRET decays with and without the addition of Nb6B9 show consistent mixing time of crude membranes (~15 s) as β2AR-Spep SPASM sensors in Fig. 1. β2AR-No peptide SPASM sensors show similar rates of decay in the presence and absence of Nb6B9. **(b)** N-acetyl-tryptophanamide (NATA) and *N*-bromosuccinimide (NBS) quenching controls used to calibrate the stopped-flow machine show increased quenching with high quencher (NBS) concentration.

Stopped flow decay curves and fitting parameters

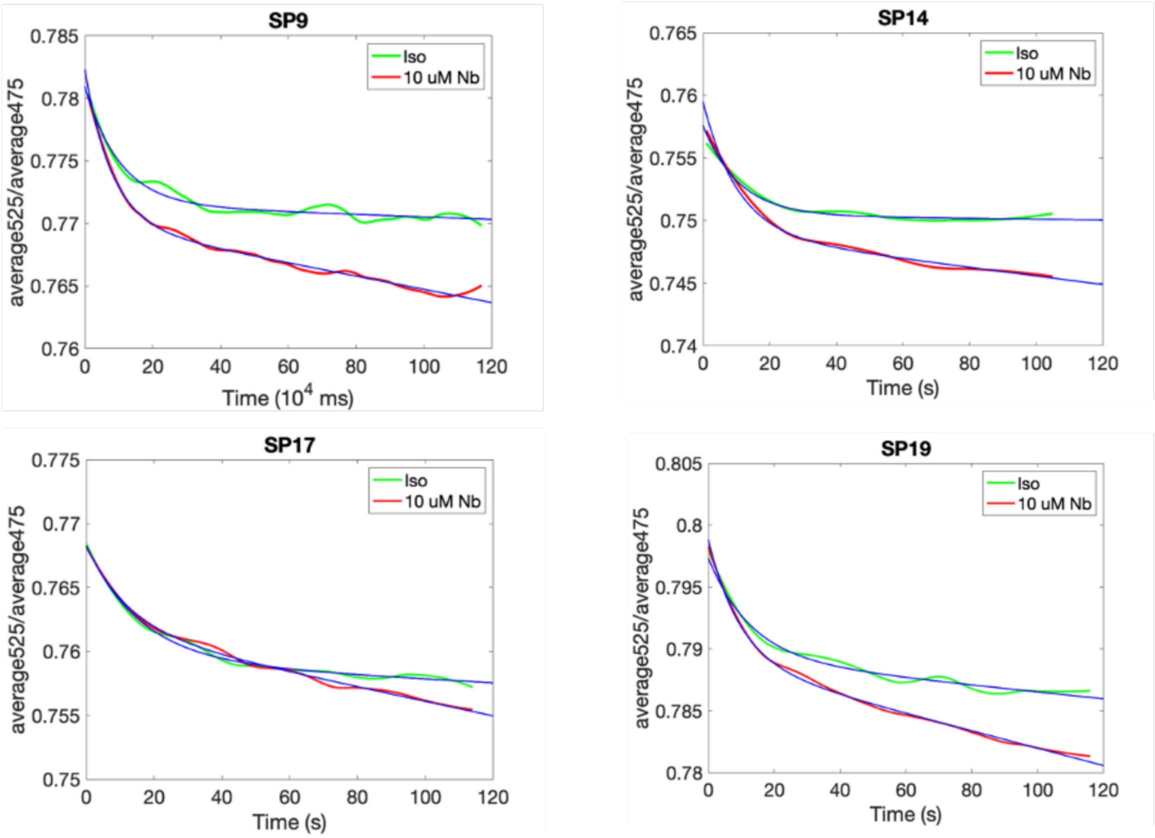

$$y = a*exp(-b*x) + c*exp(-d*x)+e*exp(-f*x)+g$$

	a<br>Fixed to iso decays	b (s-1)	c	d (s-1)	e	f (s-1)	g
S9	0.0089	0.0755	0.0051	0.0062	0.0015	0.3871	0.7585
S14	0.0066	0.0843	0.0076	0.0066	0.0022	0.1269	0.7417
S17	0.0099	0.0478	0.0024	0.0076	0.0021	0.4436	0.7579
S19	0.0101	0.0661	0.0073	0.0061	0.0015	0.2098	0.7798
Avg	0.0089	0.0684	0.0056	0.0066	0.0018	0.2919	0.7595
SD	0.0016	0.0156	0.0024	0.0007	0.0004	0.1484	0.0156
Lifetime (s)		14.6		150.9		3.4	

Supplemental Figure 7: Individual β2AR-Spep SPASM sensor decays and fitting parameters. Each decay curve shows the average counts at 525nm/the average counts at 475nm for at least 5 injections for a given membrane. Fitting parameters (table) show the slow and fast rates calculated for each batch of membranes tested (parameters b and d) as well and the rate's relative proportion of the decay curve (parameters c and e, respectively).

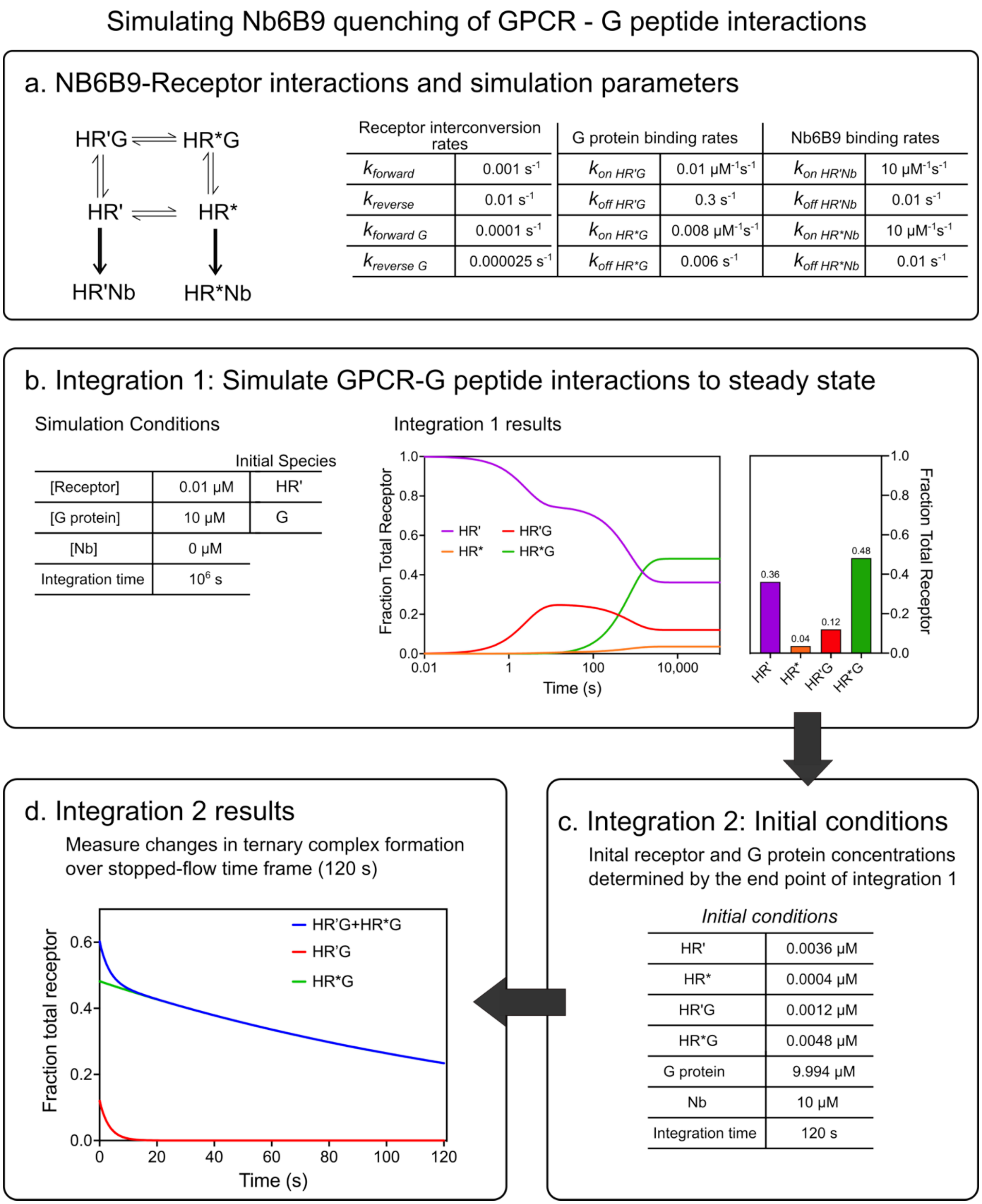

Supplemental Figure 8: Kinetic modeling for stopped flow quench experiments. (a) Parameters for G protein and nanobody (Nb) parameters **(b)** The first integration with 10 μM G protein and 0 μM Nb was run to steady-state conditions (10^6 s). The end point for each receptor species (bar graph) was stored and used as the **(c)** initial conditions for a second integration, which was run for 120 s, the length of the experiment, with 10 μM Nb **(d)** Second integration shows a decrease in the fraction of ternary complex (HR'G+HR*G). Due to slow interconversion rates, the decrease in the fraction of HR'G occurs rapidly while the decrease in HR*G occurs slowly

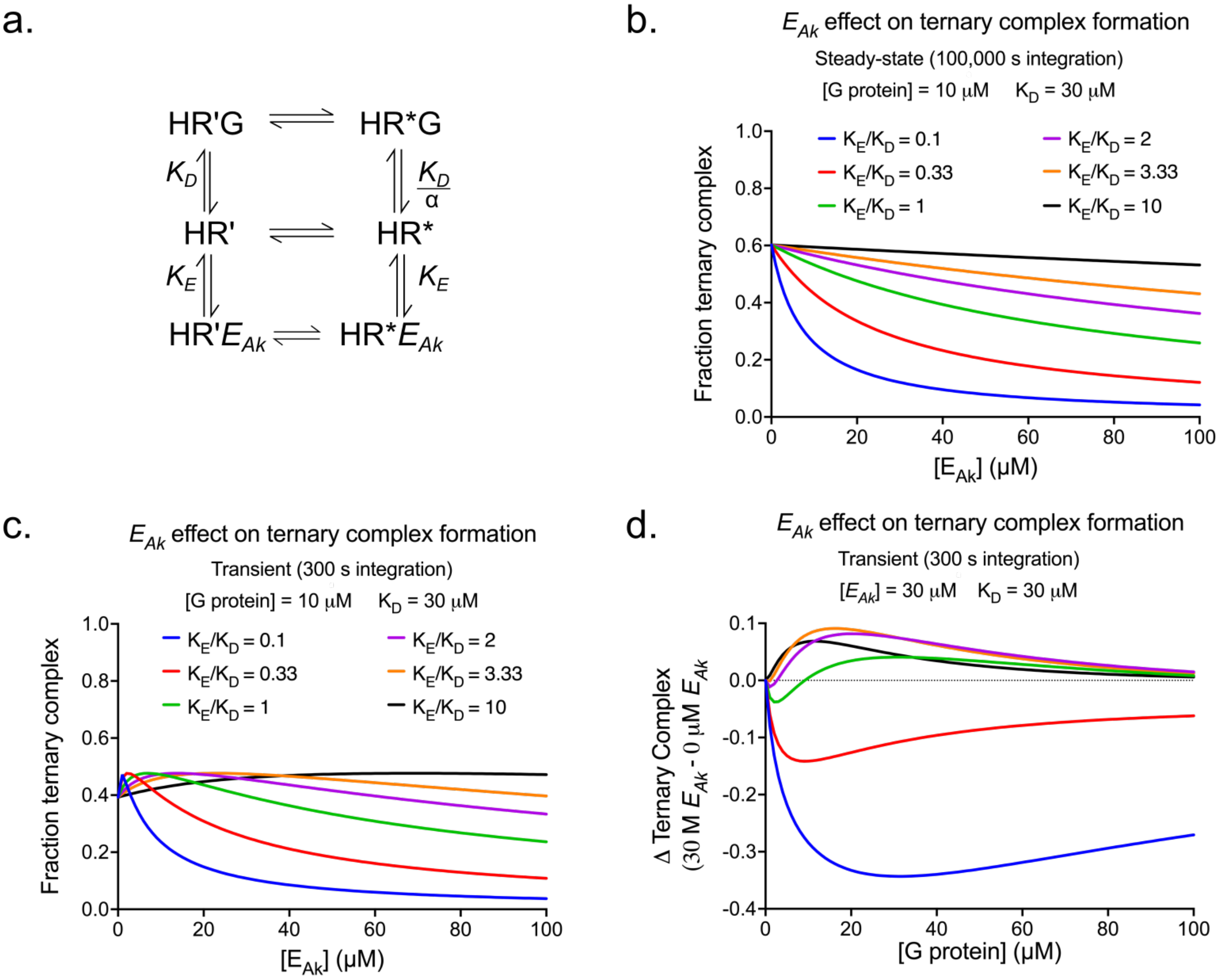

Supplemental Figure 9: *E_{Ak}* binding affinity (K_E) affects ternary complex formation (a) Kinetic modulation model shows E_{Ak} binds to HR' and HR* with the same affinity, K_E. All simulations were run using the G protein binding parameters in SFigure 5. K_E values from 3 μM to 300 μM were used and are shown in each panel as the ratio K_E/K_D **(b)** Steady state simulations show that the presence of E_{Ak} decreases ternary complex formation with 10 μM G protein at all values of K_E **(c)** Transient simulations with 10 μM G protein show that ternary complex formation is enhanced when $K_E/K_D \geq 1$ (green, purple, orange, black). **(d)** Change in ternary complex formation with 30 μM E_{Ak} shows that the greatest enhancement occurs when $10 > K_E/K_D > 1$ (orange, purple).

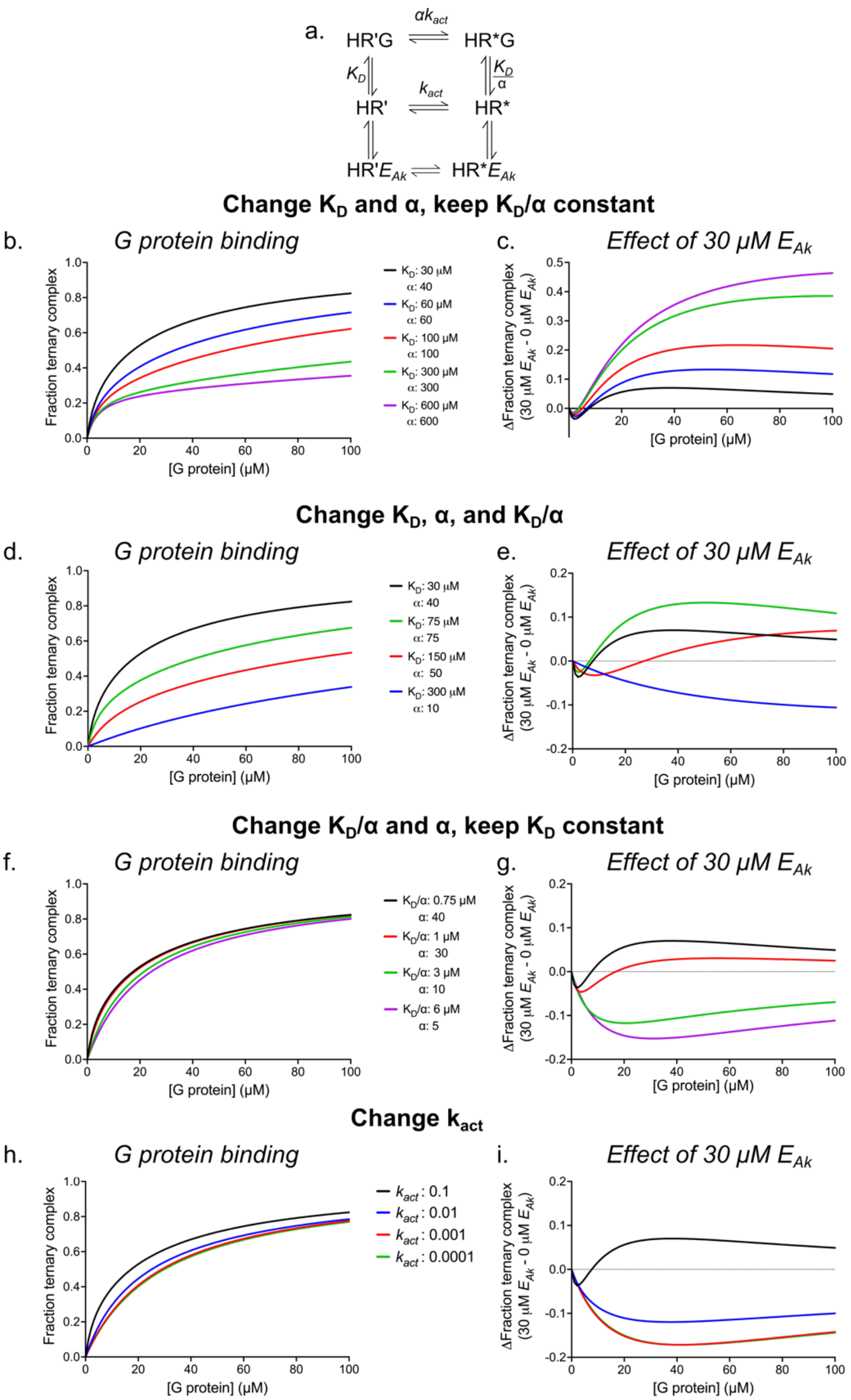

Supplemental Figure 10: Decreased binding to HR'G has the greatest effect on ternary complex formation. (b), (d), (f), (h) Ternary complex formation with specified binding and interconversion rates with no E_{Ak} present. **(c),(e),(g),(i)** Change in ternary complex formation with 30 μM E_{Ak} present under the specified binding and receptor interconversion constants. **(b),(d)** Increasing K_D and α show the greatest decrease in ternary complex formation and **(c),(e)** 30 μM E_{Ak} increases ternary complex formation with 10 μM G protein.

Supplemental Table 1: Parameters used for each simulation, by figure in main manuscript. On-rates are indicated as k.*interacting species_p* and in units of μM^{-1} s^{-1}. Off-rates are indicated as k.*interacting* species_m and in units of s^{-1}. The remaining rates are interconversion rates in s^{-1}. Initial species concentrations are in μM. Gray boxes indicate parameters that are changed from the standard simulation parameters listed in Supplemental Figure 5.

**Parameter name**		**Parameter value**				
**In code**	**In manuscript**	**Fig 1G**	**Fig 2B**	**Fig 2E**	**Fig 3C**	**Fig 4C**
R1_tot	HR' (or R or S)	0.01	0.01	0.01	0.01	0.01
Gc_tot	G (Gs or Spep or E1)	10	0 to 100	30	0 to 100	10
Gn_tot	EAk (Gq or Qpep or E2)	0, 30 **	0,10,30	0, 10	0, 30	0 to 100
R1dash	HR* (or R' or P)	0	0	0	0	0
k.Gc_R1_p	k_on_HR'G	0.01	0.01	0.01	[0.01, 0.001]	0.01
k.Gc_R1_m	k_off_HR'G	0.3	0.3	0.3	0.3	0.3
k.Gc_R1dash_p	k_on_HR*G	0.008	0.008	0.008	0.008	0.008
k.Gc_R1dash_m	k_off_HR*G	0.006	0.006	0.006	0.006	0.006
k.Gn_R1_p	k_on_HR'E	10	0.1	0.1	0.01	0.1
k.Gn_R1_m	k_off_HR'E	0.01	3	3	0.3	3
k.Gn_R1dash_p	k_on_HR*E	10	0.1	0.1	0.01	0.1
k.Gn_R1dash_m	k_off_HR*E	0.01	3	3	0.3	3
k.R1_R1dash	k_forward	0.001	0.001	0.001	0.001	0.001
k.R1dash_R1	k_reverse	0.01	0.01	0.01	0.01	0.01
k.GcR1_GcR1dash	k_forwardG	[0.0001, 0.001, 0.01, 0.1, 1]	0.001	0.001	0.001	0.001
k.GcR1dash_GcR1	k_reverseG	[0.000025, 0.00025, 0.0025, 0.025, 0.25]	0.00025	0.00025	[0.00025, 0.000025]	0.00025
k.GnR1_GnR1dash	k_forwardE	0	0.1	0.1	0.1	[0.001,0.01,0.1,1,10]
k.GnR1dash_GnR1	k_reverseE	0	1	1	1	[0.01,0.1,1,10,100]
**Binding constants**						
	alpha	40	40	40	[40,400]	40
	simulation time	** Note: No Gn initially,<br>integrate till 1e6 sec,<br>use ss values<br>as initial condition<br>for next integration<br>till 120 sec<br>where Gn is added	transient (300 sec)	transient (300 sec)	transient (300 sec)	transient (300 sec)
	KD	30	30	30	[30,300]	30
	KD/alpha	0.75	0.75	0.75	0.75	0.75
	KE	0.001	30	30	30	30
	kforwardE / kreverseE	0	0.1	0.1	0.1	0.1
	Kact	0.1	0.1	0.1	0.1	0.1

Supplemental Table 2: Parameters used for each simulation, by figure in the supplemental materials. On-rates are indicated as k.in*teractingspecies_p* and in units of $\mu M^{-1}\ s^{-1}$. Off-rates are indicated as k.*interacting*species_m and in units of s^{-1}. The remaining rates are interconversion rates in s^{-1}. Initial species concentrations are in μM. Gray boxes indicate parameters that are changed from the standard simulation parameters listed in S Figure 5.

Parameter name		Parameter value											
**In code**	**In manuscript**	**Fig S8**	**Fig S9 B**	**Fig S9 C**	**Fig S9 D**	**Fig S10 B**	**Fig S10 C**	**Fig S10 D**	**Fig S10 E**	**Fig S10 F**	**Fig S10 G**	**Fig S10 H**	**Fig S10 I**
R1_tot	HR' (or R or S)	0.01	0.01	0.01	0.01	0.01	0.01	0.01	0.01	0.01	0.01	0.01	0.01
Gc_tot	G (Gs or Spep or E1)	10	10	10	0 to 100	0 to100	0 to100	0 to100	0 to100	0 to100	0 to100	0 to100	0 to100
Gn_tot	EAk (Gq or Qpep or E2)	0, 10 **	0 to 100	0 to 100	0, 30	0	0, 30	0	0, 30	0	0, 30	0	0, 30
R1dash	HR* (or R' or P)	0	0	0	0	0	0	0	0	0	0	0	0
k.Gc_R1_p	k_on_HR'G	0.01	0.01	0.01	0.01	adjusted as per KD value	adjusted as per KD value	adjusted as per KD value	adjusted as per KD value	adjusted as per KD value	adjusted as per KD value	0.01	0.01
k.Gc_R1_m	k_off_HR'G	0.3	0.3	0.3	0.3	0.3	0.3	0.3	0.3	0.3	0.3	0.3	0.3
k.Gc_R1dash_p	k_on_HR*G	0.008	0.008	0.008	0.008	0.008	0.008	adjusted as per KD and alpha	adjusted as per KD and alpha	adjusted as per KD and alpha	adjusted as per KD and alpha	0.008	0.008
k.Gc_R1dash_m	k_off_HR*G	0.006	0.006	0.006	0.006	adjusted as per KD and alpha	adjusted as per KD and alpha	0.006	0.006	0.006	0.006	0.006	0.006
k.Gn_R1_p	k_on_HR'E	10	0.01	0.01	0.01	0.1	0.1	0.1	0.1	0.1	0.1	0.1	0.1
k.Gn_R1_m	k_off_HR'E	0.01	0.3 (varied as per KE)	0.3 (varied as per KE)	0.3 (varied as per KE)	3	3	3	3	3	3	3	3
k.Gn_R1dash_p	k_on_HR*E	10	0.01	0.01	0.01	0.1	0.1	0.1	0.1	0.1	0.1	0.1	0.1
k.Gn_R1dash_m	k_off_HR*E	0.01	0.3 (varied as per KE)	0.3 (varied as per KE)	0.3 (varied as per KE)	3	3	3	3	3	3	3	3
k.R1_R1dash	k_forward	0.001	0.001	0.001	0.001	0.001	0.001	0.001	0.001	0.001	0.001	adjusted as per Kact	0.001
k.R1dash_R1	k_reverse	0.01	0.01	0.01	0.01	0.01	0.01	0.01	0.01	0.01	0.01	0.01	adjusted as per Kact
k.GcR1_GcR1dash	k_forwardG	0.0001	0.001	0.001	0.001	0.001	0.001	adjusted as per alpha value	adjusted as per alpha value	0.001	0.001	0.0001	0.0001
k.GcR1dash_GcR1	k_reverseG	0.000025	0.00025	0.00025	0.00025	adjusted as per alpha value	adjusted as per alpha value	0.00025	0.00025	adjusted as per alpha value	adjusted as per alpha value	adjusted as per Kact	adjusted as per Kact
k.GnR1_GnR1dash	k_forwardE	0	0.1	0.1	0.1	0.1	0.1	0.1	0.1	0.1	0.1	0.1	0.1
k.GnR1dash_GnR1	k_reverseE	0	1	1	1	1	1	1	1	1	1	adjusted as per Kact	adjusted as per Kact
**Binding constants**													
	alpha	40	40	40	40	[30,60,100,300,600]	[30,60,100,300,600]	[40,75,50,10]	[40,75,50,10]	[40,30,10,5]	[40,30,10,5]	40	40
	simulation time	** Note: No Gn initially, integrate till 1e6 sec, use ss values as initial condition for next integration till 120 sec where Gn is added	steady state (1e5 sec)	transient (300 sec)	transient (300 sec)	transient (300 sec)	transient (300 sec)	transient (300 sec)	transient (300 sec)	transient (300 sec)	transient (300 sec)	transient (300 sec)	transient (300 sec)
	KD	30	30	30	30	[30,60,100,300,600]	[30,60,100,300,600]	[30,75,150,300]	[30,75,150,300]	30	30	30	30
	KD/alpha	0.75	0.75	0.75	0.75	1	1	[0.75,1,3,30]	[0.75,1,3,30]	[0.75,1,3,6]	[0.75,1,3,6]	0.75	0.75
	KE	0.001	[3,9.9,30,60,99.9,300]	[3,9.9,30,60,99.9,300]	[3,9.9,30,60,99.9,300]	30	30	30	30	30	30	30	30
	kforwardE / kreverseE	0	0.1	0.1	0.1	0.1	0.1	0.1	0.1	0.1	0.1	adjusted as per Kact	adjusted as per Kact
	Kact	0.1	0.1	0.1	0.1	0.1	0.1	0.1	0.1	0.1	0.1	[0.1,0.01, 0.001,0.0001]	[0.1,0.01, 0.001,0.0001]
			KE/KD = [0.1,0.33, 1,2,3.33,10]	KE/KD = [0.1,0.33, 1,2,3.33,10]	KE/KD = [0.1,0.33, 1,2,3.33,10]								
