# Supplementary material for: Kinetic model of GPCR-G protein interactions reveals allokairic modulation of signaling
Source: Nat Commun. 2022 Mar 8;13:1202. doi: 10.1038/s41467-022-28789-5 (PMC8904551; doi:10.1038/s41467-022-28789-5)
Supplement: Supplementary file 2 — Reporting Summary [file 41467_2022_28789_MOESM2_ESM.pdf]

nature portfolio

Corresponding author(s): Sivaraj Sivaramakrishnan, Chetan Gadgil

Last updated by author(s): 2022/01/21

Reporting Summary

Nature Portfolio wishes to improve the reproducibility of the work that we publish. This form provides structure for consistency and transparency in reporting. For further information on Nature Portfolio policies, see our Editorial Policies and the Editorial Policy Checklist.

Statistics

For all statistical analyses, confirm that the following items are present in the figure legend, table legend, main text, or Methods section.

n/a	Confirmed	
☐	☒	The exact sample size (*n*) for each experimental group/condition, given as a discrete number and unit of measurement
☐	☒	A statement on whether measurements were taken from distinct samples or whether the same sample was measured repeatedly
☐	☒	The statistical test(s) used AND whether they are one- or two-sided<br>*Only common tests should be described solely by name; describe more complex techniques in the Methods section.*
☒	☐	A description of all covariates tested
☐	☒	A description of any assumptions or corrections, such as tests of normality and adjustment for multiple comparisons
☐	☒	A full description of the statistical parameters including central tendency (e.g. means) or other basic estimates (e.g. regression coefficient) AND variation (e.g. standard deviation) or associated estimates of uncertainty (e.g. confidence intervals)
☐	☒	For null hypothesis testing, the test statistic (e.g. *F*, *t*, *r*) with confidence intervals, effect sizes, degrees of freedom and *P* value noted<br>*Give P values as exact values whenever suitable.*
☒	☐	For Bayesian analysis, information on the choice of priors and Markov chain Monte Carlo settings
☒	☐	For hierarchical and complex designs, identification of the appropriate level for tests and full reporting of outcomes
☒	☐	Estimates of effect sizes (e.g. Cohen's *d*, Pearson's *r*), indicating how they were calculated

Our web collection on statistics for biologists contains articles on many of the points above.

Software and code

Policy information about availability of computer code

Data collection	Computational calculations were performed in Matlab R2020a, using the equations and parameters outlined in the supplemental materials.
Data analysis	Figure preparation, data and statistical analysis was completed using Prism 19

For manuscripts utilizing custom algorithms or software that are central to the research but not yet described in published literature, software must be made available to editors and reviewers. We strongly encourage code deposition in a community repository (e.g. GitHub). See the Nature Portfolio guidelines for submitting code & software for further information.

Data

Policy information about availability of data

All manuscripts must include a data availability statement. This statement should provide the following information, where applicable:

- Accession codes, unique identifiers, or web links for publicly available datasets
- A description of any restrictions on data availability
- For clinical datasets or third party data, please ensure that the statement adheres to our policy

The data generated in this study is available in the Source Data File provided with this paper. Entries in the Bionumbers database were referenced in confirming parameter availability (ID: 1030809)

Field-specific reporting

Please select the one below that is the best fit for your research. If you are not sure, read the appropriate sections before making your selection.

☒ Life sciences ☐ Behavioural & social sciences ☐ Ecological, evolutionary & environmental sciences

For a reference copy of the document with all sections, see nature.com/documents/nr-reporting-summary-flat.pdf

Life sciences study design

All studies must disclose on these points even when the disclosure is negative.

Sample size	For each experiment, a single n was performed with a membrane preparation derived from one batch of cells or one passage of cells. deltaFRET measurements on a single membrane preparation (n=1)) were repeated 4 times (average of 4 measurements of agonist - 4 measurements of buffer). For a given membrane preparation (n=1), stopped flow decay curves show the decrease in FRET (525 nm/475nm) over time. To measure the FRET, the counts at 525 nm or 475 nm from at least 5 injections were averaged. For cAMP assays, one experiment (n=1) is the average of 4 data points for a given condition.
Data exclusions	Data was not excluded.
Replication	All experimental data was replicated at least 3 times. Computational calculations were performed independently by two of the authors to confirm computational calculations.
Randomization	Membranes were randomly assigned to the agonist treated and buffer treated groups.
Blinding	Experiments were not blinded due to the technical nature of the experimental protocols and the limited number of personel performing experiments it was not possible for one person to prepare samples and a second to run samples blinded.

Reporting for specific materials, systems and methods

We require information from authors about some types of materials, experimental systems and methods used in many studies. Here, indicate whether each material, system or method listed is relevant to your study. If you are not sure if a list item applies to your research, read the appropriate section before selecting a response.

Materials & experimental systems

n/a	Involved in the study	
☒	☐	Antibodies
☐	☒	Eukaryotic cell lines
☒	☐	Palaeontology and archaeology
☒	☐	Animals and other organisms
☒	☐	Human research participants
☒	☐	Clinical data
☒	☐	Dual use research of concern

Methods

n/a	Involved in the study	
☒	☐	ChIP-seq
☒	☐	Flow cytometry
☒	☐	MRI-based neuroimaging

Eukaryotic cell lines

Policy information about cell lines

Cell line source(s)	Hek293T-Flp-In were obtained from ThermoFischer (catalog number R78007)
Authentication	Cell line was not authenticated
Mycoplasma contamination	Cell line was not tested for Mycoplasma contamination
Commonly misidentified lines (See ICLAC register)	No commonly misidentified cell lines were used.
